# Thiadiazolobenzotriazole-Based Donor–Acceptor Terpolymers That Can Be Processed from Green Solvents and Deliver 950 nm Emission in Light-Emitting Electrochemical Cells

**DOI:** 10.1021/acs.chemmater.5c00984

**Published:** 2025-10-19

**Authors:** Shi Tang, Tadele T. Filate, Zewdneh Genene, Krzysztof Kotewicz, Leandro R. Franco, Qiaonan Chen, Christian Larsen, Wendimagegn Mammo, Ergang Wang, Ludvig Edman

**Affiliations:** † The Organic Photonics and Electronic Group, Department of Physics, 8075 Umeå University, Umeå SE-901 87, Sweden; ‡ lunalec AB, Umeå SE-901 87, Sweden; § Department of Chemistry and Chemical Engineering, 11248 Chalmers University of Technology, Göteborg SE-412 96, Sweden; ∥ Department of Chemistry, 37602 Addis Ababa University, PO Box 33658, Addis Ababa 1000, Ethiopia; ⊥ Department of Chemistry, Injibara University, Injibara 6040, Ethiopia; ∇ Wallenberg Initiative Materials Science for Sustainability, Department of Physics, Umeå University, Umeå SE-901 87, Sweden

## Abstract

Organic semiconductors that deliver emission with a wavelength exceeding 900 nm can enable a wide range of applications, but the unfortunate fact is that only a small number of such emitters have been synthesized and have demonstrated emissive function in devices. Here, this issue is addressed through the design and synthesis of two terpolymers that comprise a low energy-gap thiophene-thiadiazolobenzotriazole (TBTzTD) donor–acceptor unit as the minority guest incorporated in a majority donor–acceptor conjugated copolymer host, being either thiophene-quinoxaline (TQ) or thiophene-difluoroquinoxaline (TQ2F). These terpolymers are further endowed with high solubility in benign hydrophilic solvents through the grafting of branched oligo­(ethylene glycol) side chains onto the quinoxaline unit. The application function of the terpolymer emitters is demonstrated through their implementation in light-emitting electrochemical cells (LECs). It is notable from a sustainability perspective that the emitter is metal free and that the single-layer LEC active material is cast from an environmentally benign water:ethanol solvent blend. From an application perspective, it is attractive that the terpolymer-LEC devices feature a very fast turn-on to significant radiance at low voltage and that they deliver emission with a peak wavelength of 935 nm with TQ-TBTzTD as the emitter and 950 nm with TQ2F-TBTzTD as the emitter.

## Graphical abstract

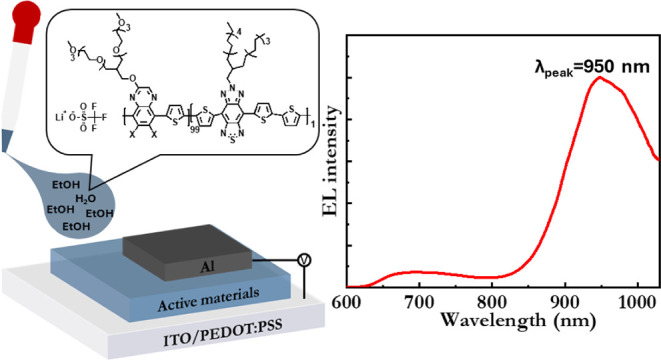

## Introduction

The development of sustainable, cost-efficient, and flexible light-emission technologies is important, not the least in the context of an increasing human population and the anticipated integration and growth of emissive technologies in the emerging fields of wearable electronics^1^ and the Internet of Things.^2^ In a sustainability context, organic light-emitting devices, such as the organic light-emitting diode (OLED) and the light-emitting electrochemical cell (LEC), can represent a good fit, since their constituent organic materials can be free from toxic and precious elements and even be sourced from abundant and renewable biomass.^3–8^ The LEC is distinguished from the OLED by that it contains mobile ions in its emissive active material, and it is the initial redistribution of these mobile ions that causes the unique and complex operational mechanism of LECs,^9–11^ enabling LEC devices to be fabricated, in their entirety, by cost-efficient ambient-air printing and coating.^12^ It has further recently been reported that the visible emission performance of LECs can be quite impressive.^13–24^

Light-emitting devices that deliver near-infrared (NIR) emission with a wavelength exceeding 900 nm are of interest for a wide range of applications, including bioimaging,^25,26^ phototheranostics,^27^ communication,^28,29^ and sensing.^30^ Table 1 presents a summary of the performance metrics of organic light-emitting devices that deliver emission above 900 nm. In brief summary, Bronstein et al. reported the synthesis of a donor–acceptor dyad emitter that featured thermally activated delayed fluorescence (TADF), and they also showed that an OLED based on this compound as the emitter delivered emission with a peak wavelength of 904 nm.^31^ Qiao and coworkers subsequently modified the well-known TADF emitter, 3-(4-(diphenylamino)­phenyl)­acenaphtho­[1,2-*b*]­pyrazino­[2,3-*e*]­pyrazine-9,10-dicarbonitrile (TPAAZ), by introducing an electron-deficient pyrazine ring, and reported that an OLED based on this emitter delivered an emission peak wavelength of 1010 nm.^32^ Ma et al. synthesized a series of small-molecule emitters with a donor−π–acceptor−π–donor architecture that featured emission above 900 nm in OLEDs.^33^ Finally, Adachi et al. synthesized a number of Yb- and Er-complexes that were utilized as the emitters in OLEDs that delivered a peak wavelength of 1000 and 1530 nm respectively,^34,35^ while Chou et al. introduced a Pt-complex as the emitter in an OLED that delivered a peak wavelength of 930 nm.^36^

**Table 1:** Survey of the OLED and LEC Devices That Emit with a Peak Wavelength above 900 nm

Emitter material	Ink solvent (if applicable and disclosed)	EL peak wavelength (nm)	EQE^a^ (%)	Peak radiance^a^ (μW/cm^2^)	ref.
Vacuum-processed OLED					
Organic small molecule	NA	904	0.019	100	^31^
Organic small molecule	NA	906	0.103		^49^
Pt complex	NA	930	2.14	4224	^36^
Yb complex	NA	1000	0.15		^34^
Organic small molecule	NA	1010	0.003	10	^32^
Organic small molecule	NA	1080	0.28	63	^33^
Er complex	NA	1530	---	---	^35^
Solution-processed OLED					
Organic copolymer	toluene	909	0.01	20	^38^
		930	0.04	1	
Organic copolymer	chlorobenzene	955	0.046	4	^39^
Zn complex	xylene	960	0.024	24	^40^
Organic copolymer	chloroform	970	0.04		^41^
Organic copolymer	chloroform	990	0.018	5.8	^42^
Organic small molecule	chloroform	1040	0.033	356	^43^
		1175	0.0025	44	
Organic small molecule	chloroform	945	0.33	2730	^44^
		1080	0.12	1220	
		1110	0.13	1240	
Solution-processed LEC					
Zn complex	chlorobenzene	900	0.028	36	^48^
Ionic Ru complex	acetonitrile	945	0.03	0.5	^47^
Organic copolymer	water:ethanol	935	0.006	20	**This work**
		950	0.026	72	

a The peak radiance and the peak EQE were not measured simultaneously.

However, a drawback from a cost and sustainability perspective is that all of the above OLED devices were fabricated by expensive and energy-intensive vapor deposition under high vacuum,^37^ and several of the emitters comprised expensive rare-earth or Pt elements that originate from environmentally problematic mining. In order to address these problems, substantial efforts have recently been directed toward the development of solution-processable and metal-free emitter compounds,^38–44^ although it should be noted that these emitters commonly are processed from toxic and environmentally problematic halogenated and/or nonhalogenated aromatic solvents.^45^

A similar cost and sustainability argument can be used to motivate why the LEC technology, with its demonstrated capacity for cost- and energy-efficient printing fabrication, is of interest.^46^ However, Table 1 reveals that only a limited number of LEC devices that emit at wavelengths exceeding 900 nm have been reported. Wang et al. synthesized a set of Ru-based emitters, which delivered light emission with a peak wavelength of 945 nm in an LEC,^47^ while Wang and coworkers developed a star-shaped diketopyrrolopyrrole-Zn-porphyrin compound and combined it with a PBDTSi-BDD host for the attainment of a peak emission wavelength of 900 nm in an LEC.^48^

Here, we report on the design and synthesis of two metal-free deep-NIR emitters that can be processed from benign solvents. The minority guest unit was designed to be thiophene-4,8-bis­(thiophene-2-yl)-6-(2-octyldodecyl)-2,1,3-thiadiazolo­[3,4-*f*]­benzotriazole (TBTzTD), which was incorporated at 1 mol % concentration into either a thiophene-quinoxaline (TQ) or a thiophene-difluoroquinoxaline (TQ2F) majority host copolymer. The resulting two terpolymers, **TQ-TBTzTD** and **TQ2F-TBTzTD**, were further endowed with branched oligo­(ethylene glycol) side chains grafted onto the quinoxaline unit, which rendered them highly soluble in benign hydrophilic solvents, such as a water:ethanol blend. We finally fabricated LEC devices by coating the single-layer active material from a benign water:ethanol ink, and we show that such ecofriendly solvent-processed LEC devices delivered NIR emission with a peak wavelength of 935 nm with **TQ-TBTzTD** being the emitter and 950 nm with **TQ2F-TBTzTD** as the emitter.

## Results and Discussion

### Molecular Design and Synthesis

The combination of an electron-rich donor (D) unit with an electron-deficient acceptor (A) unit in a D–A conjugated copolymer can result in the formation of an intramolecular charge transfer (CT) state with small energy gap and concomitant low-energy emission.^50^ Thiophene-quinoxaline (TQ)-based copolymers, with the electron-rich thiophene D unit covalently connected to the electron-deficient quinoxaline A unit, have proven particularly successful in this regard, and such copolymers have also demonstrated functionality in devices.^51–53^

In our quest for the attainment of even lower energy emission in the deep-NIR range, we hypothesize that the incorporation of designed low energy-gap guest units with quinoidal resonance could further decrease the energy gap of TQ copolymers (see Figure 1 for molecular structures). The selection of this quinoidal structure, however, necessitates careful consideration in order to tune its frontier molecular orbitals (FMOs) for efficient energy transfer and low-energy emission, and to make sure that its shallower FMOs do not result in spontaneous autoxidation of the emitter when exposed to air.^54–57^ For instance, electron-rich quinoidal polymers, such as polyisothianaphthene (PITN),^58^ with a high-lying highest occupied molecular orbital (HOMO), have proven sensitive to autoxidation and instability in air.^59^ We also wish to mention that the chemical covalent connection of the host and guest units within a single polymer is anticipated to result in a more stable film morphology, and thereby better emission performance in devices, than is attainable with the more conventional host:guest physical blending approach.^60–62^

**Figure 1:**
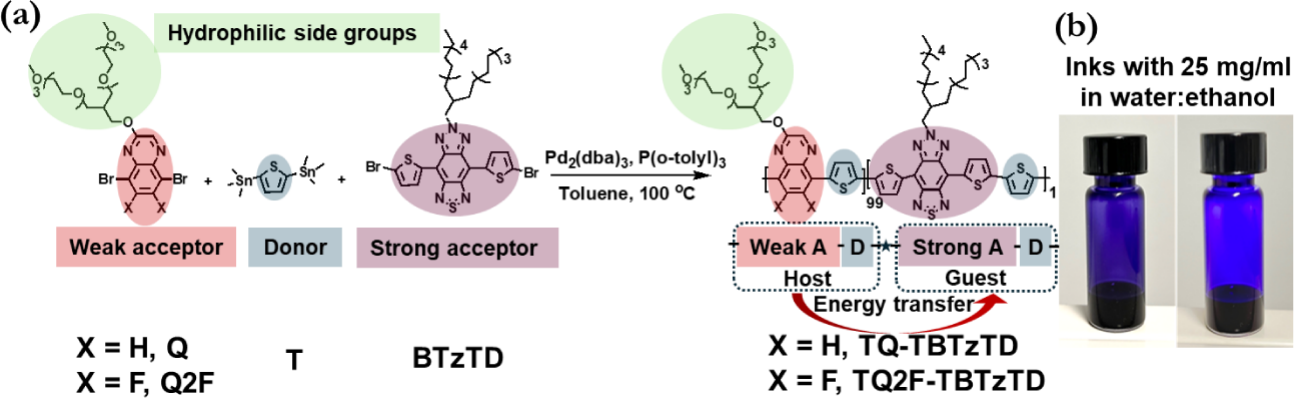
(a) The design strategy and the key steps in the synthesis of the two terpolymers **TQ-TBTzTD** and **TQ2F-TBTzTD**. (b) Photographs of inks comprising **TQ-TBTzTD** (left) and **TQ2F-TBTzTD** (right) dissolved in water:ethanol (15:85 volume ratio; solute concentration = 25 g L^–1^) under ambient illumination.

We selected to utilize an electron-deficient BTzTD moiety, a hybrid of 2*H*-benzo­[*d*]­[1,2,3]­triazole (BTz) and 2,1,3-benzothiadiazole (BTD), with demonstrated stability in air, as the strong electron-acceptor component of the guest unit.^63^ Figure S1 presents the FMOs and the molecular structure of the TQ and TQ2F host polymers and the FMOs of the BTzTD acceptor unit, with the FMOs being derived from electrochemical measurements. The FMO data indicate that TQ, TQ2F, and BTzTD exhibit sufficiently deep HOMO levels to inhibit nondesired autoxidation in air. BTzTD was combined with a thiophene D unit for the formation of a TBTzTD D–A unit, which was selected to be the minority guest to be incorporated into the TQ/TQ2F majority host for the realization of the two low energy-gap terpolymers, as displayed in Figure 1.

Furthermore, if an emissive organic semiconductor is to enable cost-efficient fabrication of devices by printing and coating, it needs to be dissolved or dispersed in solvents for the formulation of inks.^64^ During the large-scale printing and coating fabrication of commercial devices, the ink solvent is often evaporated in an open environment. Consequently, it is fundamentally important that the ink solvent is acceptable from health, safety, and environmental perspectives.^45^ Unfortunately, organic semiconductors are commonly only significantly soluble in toxic and environmentally problematic halogenated or aromatic hydrophobic solvents. However, the introduction of hydrophilic oligo­(ethylene glycol) (OEG) solubilizing groups has recently been demonstrated to be an effective strategy for the attainment of high solubility of organic semiconductors in more benign and ecofriendly hydrophilic solvents,^65–71^ and we have therefore opted to design our terpolymers with branched solubilizing OEG groups, comprising two triethylene glycol monomethyl ether units covalently linked to the quinoxaline moiety of the host copolymer.

Figure 1a presents the chemical structures of the two terpolymers **TQ-TBTzTD** and **TQ2F-TBTzTD**, along with the key steps that constituted their synthesis. Both terpolymers feature the same D–A backbone structure, but **TQ2F-TBTzTD** is distinguished by the presence of two fluorine atoms on the quinoxaline moiety, which have been reported to enhance the backbone planarity and the electron accepting capability of quinoxaline.^72^ The OEG-grafted dibromoquinoxaline acceptor monomers (Q and Q2F) were synthesized following a reported protocol,^69^ while the 4,8-bis­(5-bromothiophen-2-yl)-6-(2-ethylhexyl)-[1,2,5]­thiadiazolo­[3,4-*f*]­benzotriazole (BTzTD) acceptor monomer is commercially available. The two terpolymers were synthesized by Stille polymerization, using a 2,5-bis­(trimethylstannyl)­thiophene donor monomer and two dibromo-substituted acceptor monomers. We wish to mention that an alternative Pd-catalyzed direct heteroarylation polymerization (DHAP) reaction would be preferrable from an environmental perspective, but the drawback is that it is anticipated to result in the formation of polymeric defects for this particular combination of monomers.^73^ The detailed synthesis procedure is described in the Experimental Section.

The attainment of the desired terpolymer structures is confirmed by ^1^H NMR (Figure S2), ^13^C NMR (Figure S3), and ^19^F NMR (Figure S4) measurements. The detailed NMR peak assignments are presented in Figure S5 for **TQ-TBTzTD** and in Figure S6 for **TQ2F-TBTzTD**. The molecular weight of the terpolymers was determined with gel permeation chromatography (GPC) against a polystyrene standard (Figure S7), and this measurement yielded a reasonably high number-average molecular weight (*M*_n_) of 22.0 kDa for **TQ-TBTzTD** and 19.1 kDa for **TQ2F-TBTzTD**. The GPC data are discussed in more detail underFigure S7 of the Supporting Information.

Figure S8 presents a thermogravimetric analysis (TGA) investigation of the two terpolymers. The derived thermal degradation onset temperature (corresponding to 5% weight loss) was found to be 352 °C for **TQ-TBTzTD** and 360 °C for **TQ2F-TBTzTD**, which suggests that the two terpolymers should be robust toward the high temperatures that can result from Joule heating and nonradiative deactivation during the operation of electroluminescent devices, such as LECs.^74^ We further find that both terpolymers exhibit a desired high solubility in benign hydrophilic solvents, as exemplified by a demonstrated solubility of 25 g L^–1^ in a blend of water:ethanol (*v*:*v* = 15:85).

### Electronic Structure

Density functional theory (DFT) calculations were carried out at the B3LYP/6-311G­(d,p) level to investigate the molecular geometry conformation and the FMO densities and energy levels of the two host copolymers and guest units. To accurately represent the electronic and physical structure of the polymeric systems while maintaining computational feasibility, we choose to model the host copolymers with their corresponding trimeric oligomers, (TQ)_3_ and (TQ2F)_3_, as displayed in the first column in Figure 2.

**Figure 2:**
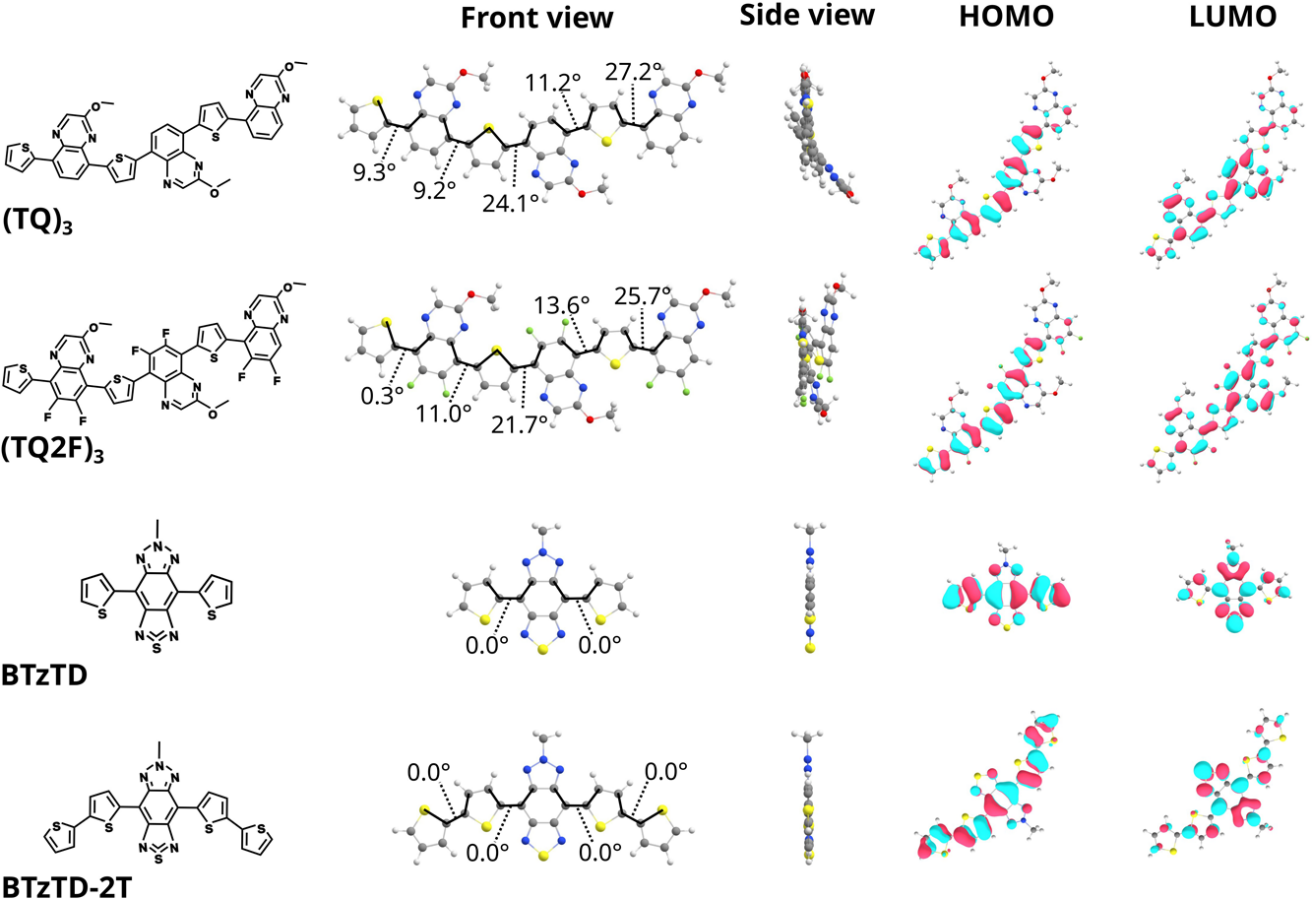
Chemical structure (first column), the optimized molecular geometry conformation in front view (second column) and side view (third column), and the HOMO and LUMO density distributions (last two columns, isovalue = 0.02) of (TQ)_3_ and (TQ2F)_3_ host trimers, BTzTD and the BTzTD-2T guest emitter.

Columns 2 and 3 present the lowest-energy conformations of these host and guest compounds, which disclose that the optimized (TQ)_3_ and (TQ2F)_3_ host geometries exhibit moderate backbone twisting, with the dihedral angles ranging between 9° and 27°. These deviations from planarity are accompanied by elongated bond lengths (∼1.46 Å) between the quinoxaline acceptor and adjacent thiophene units. These combined structural characteristics are indicative of a weakened quinoidal resonance. Although the hydrogen–fluorine (H–F) interactions in the fluorinated host slightly mitigate the backbone twisting, this conformational stabilization is less than 0.2 kcal/mol, i.e., smaller than thermal fluctuations (see Figure S9 for details). The BTzTD and BTzTD-2T guest units exhibit a much more planar conformation, with essentially zero (∼0°) dihedral angles and short bond lengths (∼1.44 Å) between the acceptor and thiophene units. These observations imply that the guests exhibit a strong quinoidal resonance. Columns 4 and 5 in Figure 2 finally show that the HOMO is, in general, well delocalized over the entire conjugated backbone, whereas the LUMO is largely localized on the electron-deficient acceptor segments.

Figure 3 presents the DFT-calculated energy levels of the FMOs of the host and guest model compounds. The fluorination of the host compound resulted in the anticipated lowering of its entire energy structure (by ∼0.2 eV). We further find that the BTzTD-2T guest exhibited the highest HOMO of −4.96 eV, whereas the HOMO of the other three compounds was relatively similar within the range of −5.16 to −5.38 eV. In contrast, both guest units featured a markedly lower LUMO by ∼0.7 eV compared to the two hosts. This implies that for the complete host–guest systems, it can be anticipated that the electron is efficiently trapped on both guest compounds, whereas the hole is trapped only on the BTzTD-2T guest. We further find that the effective energy gap of the host–guest system with BTzTD-2T as the guest compound is 1.60 eV. However, it should be mentioned that further energy-gap narrowing can be anticipated with extended conjugation.

**Figure 3:**
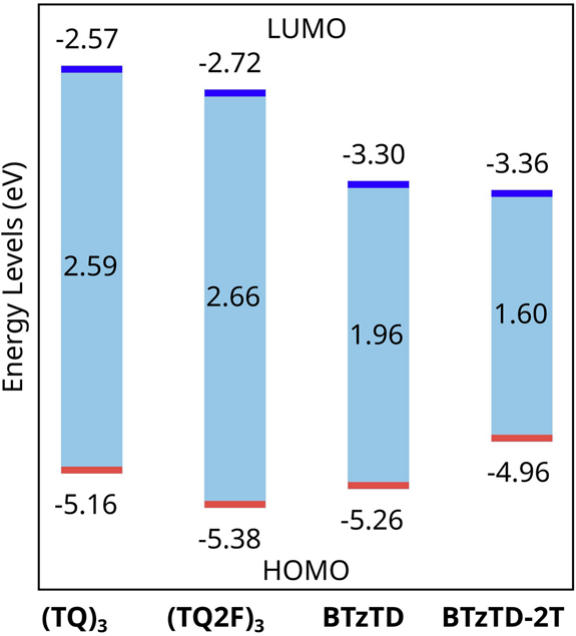
HOMO, LUMO, and energy gaps of the host and guest model compounds, as derived from DFT calculations.

### Optical and Electrochemical Properties

Figure 4a presents the normalized absorption spectrum of a dilute (1 × 10^–5^ M) solution of the guest-only BTzTD-2T compound in THF and the normalized PL spectra of neat films of the two host-only TQ and TQ2F copolymers. (The synthesis of the BTzTD-2T guest-only model compound is described in the experimental section and in Figure S10, and its ^1^H NMR and ^13^C NMR spectra are presented in Figure S11.) The observed strong spectral overlap between the emission of the host-only copolymers (solid lines) and the absorption of the guest-only model compound (dashed lines) suggests that the host-to-guest energy transfer by the Förster energy transfer (FRET) mechanism could be effective in the terpolymers. An efficient host-to-guest FRET transfer is also in line with the time-resolved PL measurements presented in Figure S12, which show that the addition of the minority guest-only compound to the host copolymer solution resulted in a marked shortening of the PL lifetime and that this fast PL emission is originating from a single fluorophore.

**Figure 4:**
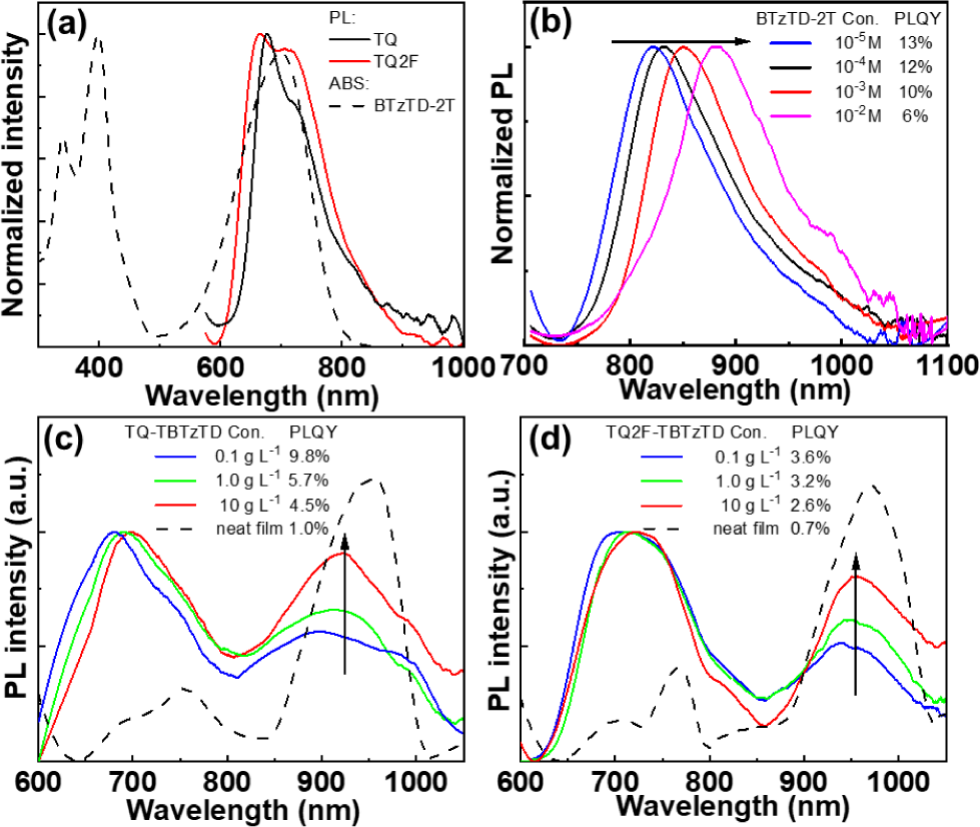
(a) The normalized absorption spectrum of a dilute (1 × 10^–5^ M) THF solution of the BTzTD-2T guest (dashed black line) and the normalized PL spectra of neat films of the TQ and TQ2F host copolymers (solid lines). (b) Evolution of the normalized PL spectrum of the BTzTD-2T guest in THF solution with increasing solute concentrations, as indicated by the arrow. The solute concentrations and the corresponding values for the PLQY are presented in the inset. (c, d) The PL spectra of the terpolymers (c) **TQ-TBTzTD** and (d) **TQ2F-TBTzTD** in a water:ethanol (*v*:*v* = 15:85) blend solution and as a neat film. The arrows indicate increasing solute concentration. The solute concentrations and corresponding PLQY values are identified in the inset. The neat films were spin-coated from a 25 g L^–1^ water:ethanol (*v*:*v* = 15:85) blend solution. The excitation wavelength in the PL measurements was 550 nm.

Figure 4b shows the evolution of the normalized PL spectrum and the PLQY (see inset) of the BTzTD-2T guest-only compound with increasing solute concentration. We find that the PL peak redshifts by 65 nm and that the PLQY drops by more than 50% when the BTzTD-2T concentration increases from 1 × 10^–5^ M to 1 × 10^–2^ M. We further find that a neat film of BTzTD-2T does not exhibit any detectable PL. We rationalize these observations by that the planar conformation of the isolated BTzTD-2T guest (as shown in Figure 2) renders it highly prone to π–π stacking, which in turn causes a redshift and quenching of its emission.

We now shift our attention to the photophysical investigation of the two terpolymers. Figure S13 presents the absorption spectra of the terpolymers in dilute (0.03 mg mL^–1^) water:ethanol (*v*:*v* = 15:85) solution and as a neat thin film. We find that both terpolymers exhibit essentially identical absorption spectra in dilute solution and as a neat film, which implies a lack of aggregation. The weak absorption peak at ∼820 nm in the two terpolymers (that is absent in the host-only copolymers) is attributed to absorption by the minority (1 mol %) chemically incorporated BTzTD-2T guest emitter.^75,76^

Figure 4c,d presents the PL spectra of the two terpolymers in water:ethanol solution at three different solute concentrations (solid lines) and as a neat film (dashed black line), with the inset identifying the corresponding values for the PLQY. In the solution, two distinct PL bands are observed, with the deep-red peak at ∼700 nm being assigned to emission by the majority host component, while the NIR peak at ∼900 nm is attributed to emission by the chemically incorporated TBTzTD guest. More specifically, a comparison with Figure 4a yields that the peak wavelength of the higher-energy deep-red band of the nonfluorinated terpolymer **TQ-TBTzTD** is almost identical to that of the host-only TQ copolymer,^69^ while the peak wavelength of the same higher-energy band is red-shifted by 25 nm for the fluorinated **TQ2F-TBTzTD** terpolymer compared to the host-only TQ2F copolymer. In this context, we note that a tendency for aggregation due to the presence of fluorine atoms has been reported for similar polymer systems.^77–79^

The relative intensity of the lower-energy NIR band compared to the higher-energy deep-red band increases with increasing terpolymer concentration (as indicated by the arrow) and is the highest in the neat film, which implies that the host-to-guest energy transfer is effectuated by a combination of intramolecular *and* intermolecular interactions. We further find that the PLQY decreases with increasing terpolymer concentration when the NIR peak becomes increasingly prominent, which is in line with the so-called energy-gap law.^80^ We have also measured the PL spectrum and the PLQY of the two terpolymers in acetone solution and in neat films cast from the acetone solvent and observe a similar behavior as with the water:ethanol blend solvent (see Figures S14 and S15).

We finally note that the **TQ-TBTzTD** and **TQ2F-TBTzTD** terpolymers feature a red-shifted NIR emission compared with the guest-only BTzTD-2T compound, which suggests that the effective conjugation length of the guest emitter in the host–guest terpolymers is increased compared to that of the guest-only model compound.

Figure S16 presents CV traces recorded on neat terpolymer thin films. The observed reversible electrochemical oxidation and reduction reactions for the two terpolymers suggest that the two terpolymers can be electrochemically both p- and n-type doped. This in turn indicates that the terpolymers could be suitable for the electroactive compound in LECs, where electrochemical n- and p-type doping are essential features during the initial operation.^81^ The HOMO and LUMO levels of the terpolymers were calculated with the following Equation:

1 $$E_{\mathrm{HOMO}/\mathrm{LUMO}}=-\left(V_{ox/red}^{onset}+5.13\right)eV$$

where $V_{ox/red}^{onset}$ is the oxidation/reduction onset potential determined in the CV measurement.

Table 2 shows that the **TQ-TBTzTD** terpolymer exhibits electrochemical HOMO and LUMO values of −5.27 and −3.61 eV, respectively, whereas the fluorinated **TQ2F-TBTzTD** features slightly lower HOMO/LUMO levels of −5.38/–3.74 eV. The observed downshift, or stabilization, of the HOMO and LUMO levels following fluorination is a common observation in organic semiconductors^72,82^ and in agreement with the DFT data in Figure 3 on the model host compounds. The electrochemical energy gap, as gleaned from the CV measurement, was however essentially identical for both terpolymers at 1.64–1.66 eV. We note that the optical energy gap, as derived from the absorption spectra displayed in Figure S13, is 0.2 eV larger than the electrochemical energy gap for both terpolymers (see Table 2).

**Table 2:** Optical and Electrochemical Properties of the Two Terpolymers

				λ_abs_ (nm)			λ_PL_ (nm)	
Terpolymer	LUMO^a^ (eV)	HOMO^a^ (eV)	*E*_g_^CV^ (eV)	solution	film	*E*_g_opt^b^ (eV)	solution	film
**TQ-TBTzTD**	–3.61	–5.27	1.66	569	573	1.86	680, 900	956
TQ2F-TBTzTD	–3.74	–5.38	1.64	556	562	1.86	705, 941	970

a Calculated from CV.

b Calculated from the low-energy absorption onset using the Tauc method.

### The Light-Emitting Electrochemical Cell

We now shift our attention to the investigation of the merit of terpolymers in devices, more specifically LECs. The terpolymers were mixed with a LiCF_3_SO_3_ salt in an optimized 100:3 mass ratio, and this mixture was dissolved with a solute concentration of 25 g L^–1^ in a water:ethanol (*v*:*v* = 15:85) blend solvent for the formulation of the active-material ink. The motivation for selecting the LiCF_3_SO_3_ salt as the source of the mobile ions is that it exhibits high solubility in a wide variety of oligo-ether solvents, akin to the oligoether side groups of the terpolymers, and because it previously has demonstrated good performance in LEC devices.^83,84^ The active-material ink was deposited on an air-stable poly­(3,4-ethylenedioxythiophene):polystyrenesulfonate (PEDOT:PSS)-coated indium tin oxide (ITO) anode by spin coating, and the device structure was completed by the thermal evaporation deposition of an air-stabilized Al cathode.

Figure 5a presents the initial transients of the voltage and radiance when the pristine terpolymer LECs were driven by a constant current density of 250 mA cm^–2^. The observed initial drop of the voltage is assigned to the mobile ions in the active material redistributing to first form injection-facilitating electric double layers at the electrode interfaces and then transport-enhancing electrochemical doping of the terpolymer, whereas the increase in radiance is due to enhanced electron and hole recombination into excitons when the doping regions have made contact and formed a p–n junction. Thus, it appears clear that both terpolymer devices are functional LECs, which feature ion redistribution and bipolar electrochemical doping.^85^

**Figure 5:**
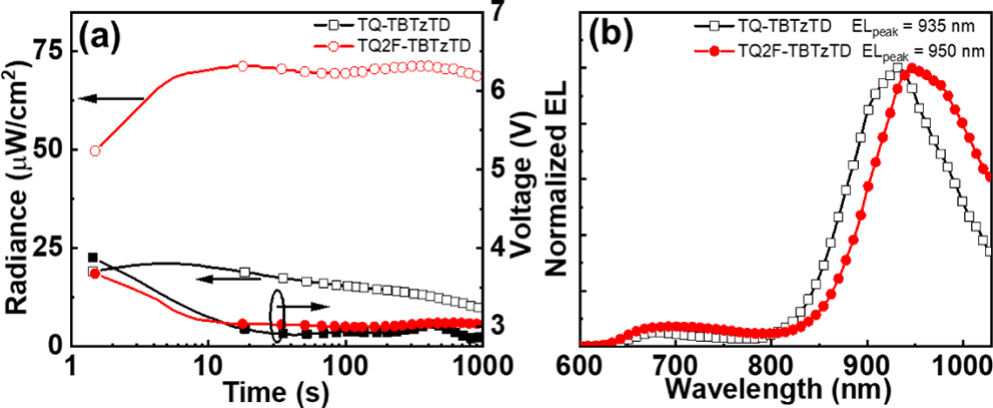
(a) The temporal evolution of the radiance (left *y*-axis) and the voltage (right *y*-axis) of LEC devices with a terpolymer as the emitter (see inset) when driven with a constant current density of 250 mA cm^–2^. (b) The normalized EL spectrum recorded at steady state for the two terpolymer-LECs.

Importantly, Figure 5b shows that both terpolymer-LECs deliver the vast majority of their emission with a wavelength above 900 nm, with the LEC based upon **TQ-TBTzTD** featuring an emission peak wavelength of 935 nm whereas the device comprising **TQ2F-TBTzTD** delivers emission with an even longer peak wavelength of 950 nm. This NIR capacity is visualized by the two photographs in Figure S17 of a **TQ2F-TBTzTD** LEC driven by 4 V, which were recorded with and without a filter that allows wavelengths larger than 800 nm to be detected. Additionally, the **TQ2F-TBTzTD** LEC also exhibited higher values for the peak radiance of 72 μW cm^–2^ and the external quantum efficiency (EQE) of 0.026% during the constant-current density operation. Interestingly, there is currently no clear trend to discern as regards to the value of introducing fluorine atoms on the emitter in LEC devices,^69,86^ and it is plausible that the fluorine effect instead is secondary in that it is the attained energy levels and mobility balance that is primarily dictating the LEC performance.^87,88^

A simple efficiency analysis, considering that the terpolymer is a singlet emitter, that the solid-state PLQY of the neat **TQ2F-TBTzTD** terpolymer is ∼0.7% (see Figure 4d), that the electron:hole recombination efficiency is effectively perfect in a p-n homojunction (that has formed at steady state in an LEC),^87,88^ and somewhat naively assuming that the outcoupling efficiency is ∼20%, yields that the exciton losses (to primarily exciton:polaron quenching interactions)^88^ are of the order of 50%. Thus, two straightforward means to improve the emission efficiency of the terpolymer LECs are to increase the solid-state PLQY and to decrease the losses to exciton:polaron quenching interactions, where a functional mean toward the latter is the inclusion of a host compound^13^ with designed energy levels and balanced electron:hole transport capacity for an appropriate widening^88^ and positioning^87^ of the p–n junction.

We finally compare our herein attained emission performance to those of previously reported LECs featuring an emission peak wavelength above 900 nm. Table 1 reveals that the **TQ2F-TBTzTD**-based device with its peak wavelength of 950 nm delivered the deepest NIR emission from an LEC so far. The emission efficiency of this device is also on par with previous deep-NIR LECs using a Zn or Ru complex as the emitter, although it is notable that the **TQ2F-TBTzTD**-based LEC delivered this emission at a significantly higher radiance than those of previously reported LECs. Moreover, Figure 5a shows that the turn-on time to a significant radiance of 50 μW cm^–2^ is very fast for this device at 1 s, and that the required drive voltage remains below 4 V during both turn-on and steady-state emission. Both terpolymer-based LECs also offer appealing properties from a sustainability perspective in that the emitter is completely metal-free and that the active material can be processed from nontoxic and ecofriendly solvents.

## Conclusions

We report on the design, synthesis, and application of two terpolymers engineered for sustainable long-wavelength NIR emission. The terpolymers comprise a low energy-gap TBTzTD donor–acceptor unit as the minority (1 mol %) guest incorporated into a majority donor–acceptor TQ or TQ2F conjugated copolymer host. The terpolymers were further designed for high solubility in benign hydrophilic solvents through the grafting of branched oligo­(ethylene glycol) side chains onto the quinoxaline host unit. The functionality of the developed terpolymers was demonstrated through their implementation as the emitter in LEC devices, which featured a very fast turn-on to a significant radiance at a low voltage and which—importantly—deliver NIR emission with a peak wavelength of 935 nm with TQ-TBTzTD being the emitter and 950 nm with TQ2F-TBTzTD. It is notable from a sustainability perspective that the developed terpolymer emitters are metal free and that the single-layer LEC active material was cast from an environmentally benign water:ethanol solvent blend. Accordingly, this study highlights a promising molecular design and device strategy for the attainment of long-wavelength NIR emission from a thin-film device that can be both low-cost and sustainable.

## Experimental Section

### Materials and Synthesis

All materials, chemicals, and solvents were purchased from Fisher Scientific, Sigma-Aldrich, and Derthon and were used as received. The detailed synthesis of the OEG-grafted quinoxaline monomers is described in our previous reports,^68,69^ and their corresponding characterization is presented in Figures S18–S20. The synthesis of the terpolymers was executed by Stille polymerization using a palladium catalyst.

#### TQ-TBTzTD

2-((13-(2,5,8,11-Tetraoxadodecyl)-2,5,8,11-tetraoxatetradecan-14-yl)­oxy)-5,8-dibromoquinoxaline (Q) (132.7 mg, 193.9 μmol), 4,8-bis­(5-bromothiophen-2-yl)-6-(2-ethylhexyl)-[1,2,5]­thiadiazolo­[3,4-*f*]­benzotriazole (BTzTD) (1.5 mg, 2.0 μmol), 2,5-bis­(trimethylstannyl)­thiophene (T) (80.2 mg, 195.8 μmol), tris­(dibenzylideneacetone)­dipalladium(0) (Pd_2_(dba)_3_) (3.6 mg, 3.9 μmol), and tri­(*o*-tolyl)­phosphine (P­(*o*-Tol)_3_) (4.8 mg, 15.7 μmol) were charged into a reaction vessel (20 mL). The mixture was degassed and filled with nitrogen 5 times. Anhydrous toluene (10 mL) was added, and the mixture was heated at 100 °C under reflux for 24 h. The polymer chains were quenched by a sequential reaction with 2-(tributylstannyl)­thiophene and 2-bromothiophene. After being cooled to room temperature, the crude polymer was precipitated from hexanes and filtered through a Soxhlet extraction thimble. Subsequently, the polymer was extracted with hexanes, diethyl ether, methanol, and chloroform. The chloroform fraction was concentrated and precipitated again from hexanes, filtered using a PTFE membrane (0.45 μm), and dried under vacuum at 40 °C overnight to afford **TQ-TBTzTD** (92 mg, 77.3%).

#### TQ2F-TBTzTD

2-((13-(2,5,8,11-Tetraoxadodecyl)-2,5,8,11-tetraoxatetradecan-14-yl)­oxy)-5,8-dibromo-6,7-difluoroquinoxaline (**Q2F**) (117.4 mg, 163.0 μmol), BTzTD (1.3 mg, 1.6 μmol), 2,5-bis­(trimethylstannyl)­thiophene (T) (67.4 mg, 164.6 μmol), tris­(dibenzylideneacetone)­dipalladium(0) (Pd_2_(dba)_3_) (3.0 mg, 3.3 μmol), and tri­(*o*-tolyl)­phosphine (P­(*o*-Tol)_3_) (4.0 mg, 13.2 μmol) were charged into a reaction vessel (20 mL). The mixture was degassed and filled with nitrogen 5 times. Anhydrous toluene (10 mL) was added, and the mixture was heated at 100 °C under reflux for 24 h. The polymer chains were quenched by a sequential reaction with 2-(tributylstannyl)­thiophene and 2-bromothiophene. After cooling to room temperature, the crude polymer was precipitated from hexanes and filtered through a Soxhlet extraction thimble. Subsequently, the polymer was extracted with hexanes, diethyl ether, methanol, and chloroform. The chloroform fraction was concentrated and precipitated again from hexanes, filtered using a PTFE membrane (0.45 μm), and dried under vacuum at 40 °C overnight to afford **TQ2F-TBTzTD** (90 mg, 85.0%).

#### BTzTD-2T

BTzTD (0.35 g, 0.45 mmol), 2-tributylstannylthiophene (0.84 g, 2.24 mmol), Pd_2_(dba)_3_ (0.017 g, 0.0256 mmol), and tri­(*o*-tolyl)­phosphine (0.022 g, 0.1024 mmol) were dissolved in anhydrous THF (30 mL) under nitrogen and heated at 80 °C for 24 h. Then, the mixture was cooled to room temperature and the crude reaction mixture was poured over distilled water and extracted with diethyl ether. The combined organic phase was washed with water (3 × 100 mL) and dried over anh. MgSO_4_. The crude product was purified by column chromatography over silica gel using 4:1 (v/v) hexane:dichloromethane as the eluent to yield BTzTD-2T (0.18 g, 51%) as a green solid. ^1^H NMR (400 MHz, CDCl_3_) δ 8.60 (*d*, *J* = 4.1 Hz, 2H), 7.35 (*d*, *J* = 3.6 Hz, 2H), 7.31 – 7.25 (*m*, 4H), 7.07 (*t*, *J* = 4.3 Hz, 2H), 4.76 (*d*, *J* = 6.4 Hz, 2H), 2.39 – 2.25 (*m*, 1H), 1.44 – 1.17 (*m*, 32H), 0.88 – 0.81 (*m*, 6H). ^13^C NMR (101 MHz, CDCl_3_) δ: 149.6, 142.3, 140.9, 137.8, 136.3, 131.9, 128.0, 124.7, 124.4, 123.9, 111.3, 60.9, 39.2, 31.9, 31.9, 31.5, 29.9, 29.7, 29.7, 29.7, 29.6, 29.4, 26.3, 22.7, 14.1.

### Emitter Characterization

The ^1^H NMR and ^13^C NMR spectra were recorded on a Varian Inova 400 MHz spectrometer at 400.13 and 100.6 MHz, respectively, whereas the polymer ^1^H NMR and ^13^C NMR spectra were recorded on a Bruker Avance Neo 600 MHz NMR spectrometer at 600 and 151 MHz, respectively. The mass spectra were obtained with a Xevo G2-XS QTof mass spectrometer equipped with electrospray ionization. The ultraviolet–visible (UV–vis) absorption spectra were measured with a PerkinElmer lambda 1050 UV/vis/NIR spectrometer. The PL spectra and the PLQY were recorded with an integrating sphere connected to a spectrometer (C9920–02G, Hamamatsu Photonics). The PL intensity transients were recorded with a pulsed laser (wavelength = 375 nm, frequency = 500 Hz) as the excitation source and a spectrometer (FLS1000, Edinburgh) for the detection. The TGA was measured with a Mettler Toledo TGA/DSC3+. The CV measurements were performed with a CH Instruments electrochemical workstation, using a terpolymer-coated Pt wire as the working electrode, Ag/AgCl as the reference electrode, and a Pt wire as the counter electrode. The terpolymers were coated on the Pt wire from a 10 g L^–1^ chloroform solution. The electrolyte solution consisted of 0.1 M tetrabutylammonium hexafluorophosphate in anhydrous acetonitrile. Ferrocene/ferrocenium ion (Fc/Fc^+^) was the internal reference. All measurements were conducted under a nitrogen atmosphere. Gel permeation chromatography (GPC) measurements were performed with an Agilent 1260 Infinity II GPC/SEC System, relative to a polystyrene standard using chloroform as the eluent at 35 °C equipped with a UV detector monitoring at a 350 nm wavelength. The surface morphology of the thin films was measured with atomic force microscopy in noncontact mode (NX-Hivac, Park Systems).

### DFT Calculations

The ground-state electronic structures of the host copolymers and guest molecules were investigated by using Density Functional Theory (DFT). Calculations employed the B3LYP exchange-correlation functional^89^ with the triple-ζ quality 6-311G­(d,p) basis set^90^ and Grimme’s dispersion correction with Becke-Johnson damping (D3-BJ),^91^ as implemented in Gaussian 16 (Rev C.01).^92^ Gibbs free energies were determined in the gas phase from harmonic frequency calculations including thermal corrections to the electronic energies. To reduce computational cost, the OEG and alkyl side chains were simplified by replacing them with methoxy and methyl groups, respectively. Molecular geometries were optimized in the gas phase, while electronic properties were calculated in ethanol solvent, modeled using the universal continuum solvation model (SMD).^93^

### Device Fabrication and Characterization

**TQ-TBTzTD** and **TQ2F-TBTzTD** were separately dissolved in a water:ethanol (*v*:*v* = 15:85) solvent blend at a solute concentration of 25 g L^–1^, and the master inks were thereafter stirred at 70 °C for >6 h on a magnetic hot plate. The active-material ink was prepared adding the LiCF_3_SO_3_ (Merck, 99%) salt to the terpolymer master inks in a terpolymer:LiCF_3_SO_3_ solute mass ratio of 100:3. The active-material inks were stirred at 70 °C for >12 h on a magnetic hot plate. The ITO-coated glass substrates (glass thickness = 0.7 mm, glass area = 30 × 30 mm^2^, *R*_S_ = 20 Ω sq^–1^, Kintec) were carefully cleaned by sequential 15 min ultrasonication in alkaline cleaning solution, acetone, and 2-propanol.^94^ A PEDOT–PSS ink (Clevios P VP AI 4083, Heraeus) was spin-coated onto the ITO surface at 4000 rpm for 60 s, and the deposited film was dried at 120 °C for 30 min. The active-material ink was spin-coated onto the PEDOT–PSS surface at 2000 rpm for 60 s and thereafter dried under vacuum for >12 h. The thickness of the PEDOT–PSS layer was 40 nm, and the thickness of the active-material layer was 100 nm, as measured with a profilometer (DekTak XT, Bruker). Finally, a set of four Al electrodes was deposited on top of the active material by thermal evaporation at *p* < 5 × 10^–4^ Pa. The light-emission area, as defined by the cathode-anode overlap, was 0.2 × 0.2 cm^2^. The LECs were driven by a constant current density, and the voltage was measured by a microcontroller (Arduino UNO) connected to a computer. The ITO electrode was invariably biased as a positive anode. The emitted radiance was measured with a calibrated Si photodiode (S2387-33R, Hamamatsu), and the emission spectrum was measured with a spectrometer (USB2000+, Ocean Optics). The photographs of LEC devices during emission were recorded with a camera using an exposure time of 5 s and an aperture of F/1.6. A removable cutoff filter (B+W IR-filter 093) was used to selectively image the emission of wavelengths larger than 800 nm. All of the above procedures, with the exception of the deposition of the PEDOT:PSS and the active-material layers, were carried out in two interconnected N_2_-filled glove boxes ([O_2_] < 1 ppm, [H_2_O] < 0.5 ppm).

## Supplementary Material



## Data Availability

The experimental data are available for download at: https://figshare.com/s/51f851773ca259da1faa

## Notes

Chemical structures and energy levels of the host copolymers; ^1^H NMR and ^13^C NMR spectra of the terpolymers, the guest compound, and key monomers; thermogravimetric analysis; gel permeation chromatography; conformational simulation; absorption spectra; PL spectra in acetone solution; photographs and atomic force microscopy images of spin-coated terpolymers films; time-resolved PL; cyclic voltammetry (PDF)
