# Supplementary material for: Thiadiazolobenzotriazole-Based Donor–Acceptor Terpolymers That Can Be Processed from Green Solvents and Deliver 950 nm Emission in Light-Emitting Electrochemical Cells
Source: Chem Mater. 2025 Oct 19;37(20):8120–30. doi: 10.1021/acs.chemmater.5c00984 (PMC12573754; doi:10.1021/acs.chemmater.5c00984)
Supplement: Supplementary file 1 [file cm5c00984_si_001.pdf]

<u>Supporting Information</u>:

Thiadiazolobenzotriazole-Based Donor-Acceptor Terpolymers that Can be Processed from Green Solvents and Deliver 950 nm Emission in Light-Emitting Electrochemical Cells

Shi Tang,[1,2,‡] Tadele T. Filate,[3,4,5,‡] Zewdneh Genene,[3] Krzysztof Kotewicz,[3] Leandro R. Franco,[3] Qiaonan Chen,[3] Christian Larsen,[1,2] Wendimagegn Mammo,[4,] Ergang Wang[3,*] and Ludvig Edman,[1,2,6,*]*

[1]The Organic Photonics and Electronic Group, Department of Physics, Umeå University, SE-901 87 Umeå, Sweden.

[2]LunaLEC AB, SE-901 87, Umeå, Sweden.

[3]Department of Chemistry and Chemical Engineering, Chalmers University of Technology, SE-412 96 Göteborg, Sweden.

[4]Department of Chemistry, Addis Ababa University, PO Box 33658, 1000 Addis Ababa, Ethiopia.

[5]Department of Chemistry, Injibara University, 6040 Injibara, Ethiopia

[6]Wallenberg Initiative Materials Science for Sustainability, Department of Physics, Umeå University, SE-901 87 Umeå, Sweden.

‡S.T. and T.T.F. contributed equally to this work.

*E-mail: wendimagegn.mammo@aau.edu.et (W.M.)

*E-mail: ergang@chalmers.se (E.W.)

*E-mail: ludvig.edman@umu.se (L.E.)

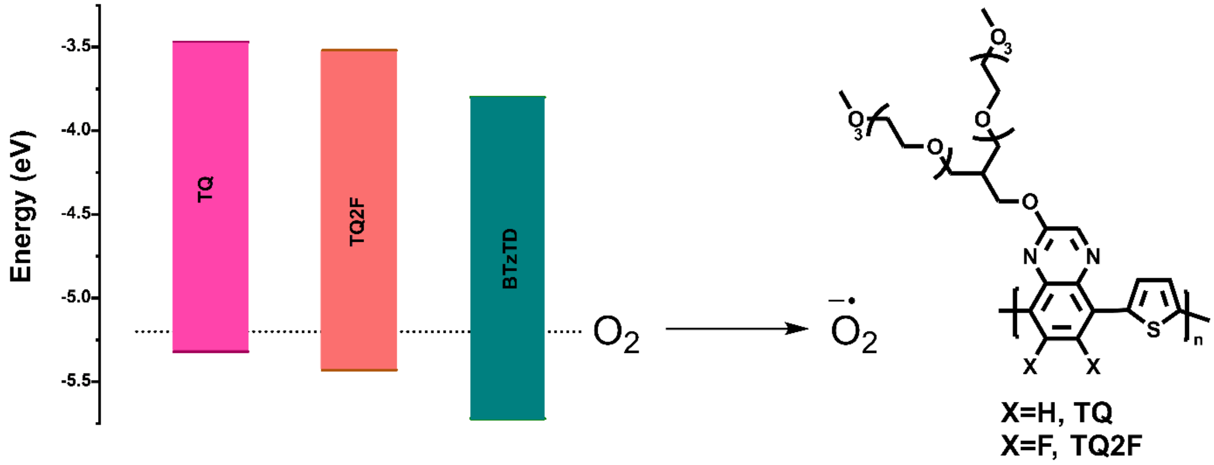

Figure S1. (left) The energy levels of the FMOs of the two TQ copolymers and the BTzTD unit, as derived from CV measurements.[1, 2] The tabulated first reduction potential of the dioxygen molecule is included as reference.[3] (right) The molecular structure of the two host-only polymers.

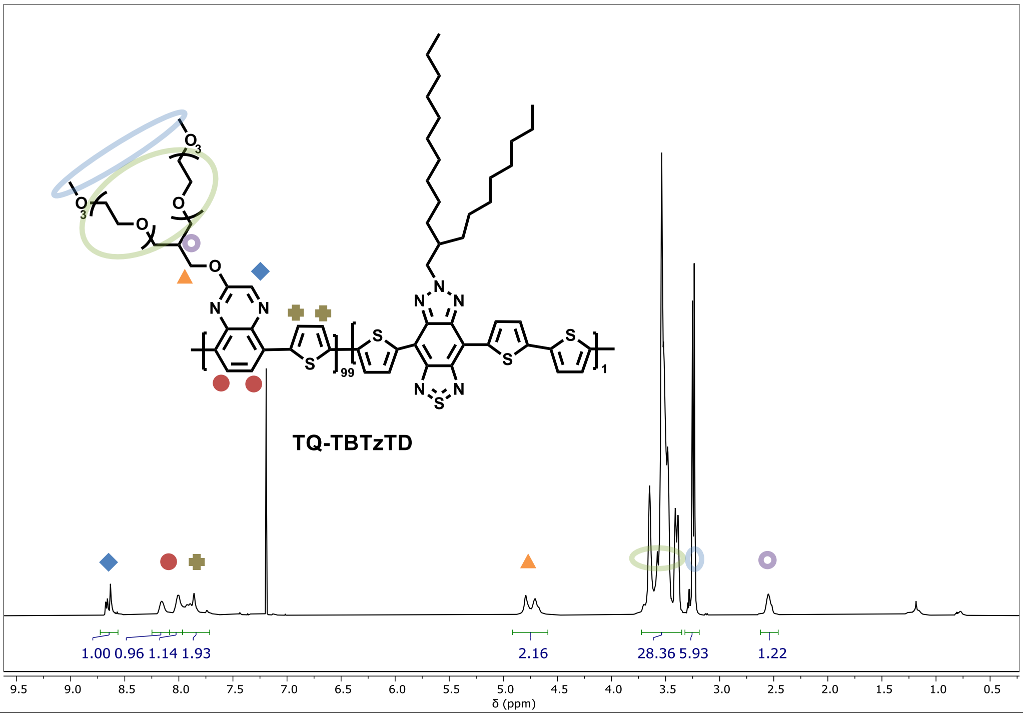

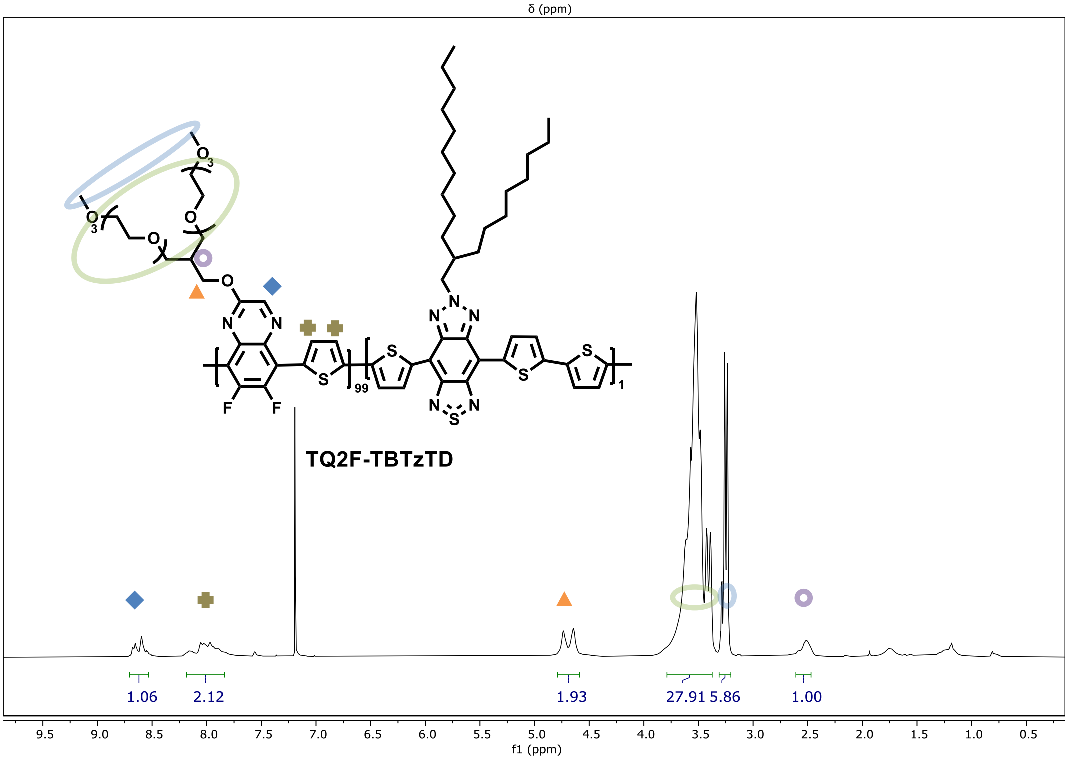

Figure S2. The ^{1}H NMR spectra of (upper) **TQ-TBTzTD** and (lower) **TQ2F-TBTzTD**.

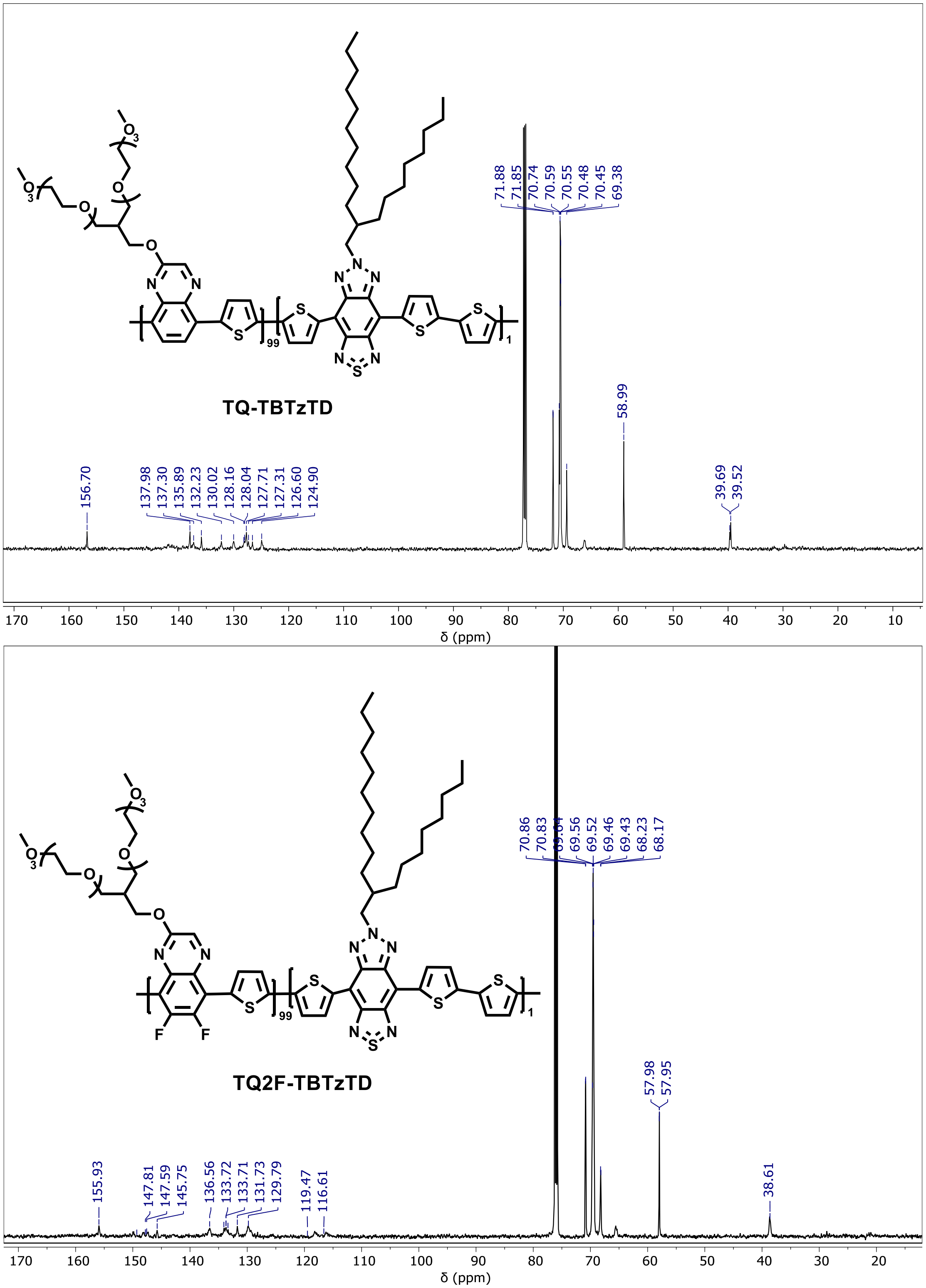

Figure S3. The ^{13}C NMR spectra of (upper) **TQ-TBTzTD** and (lower) **TQ2F-TBTzTD**.

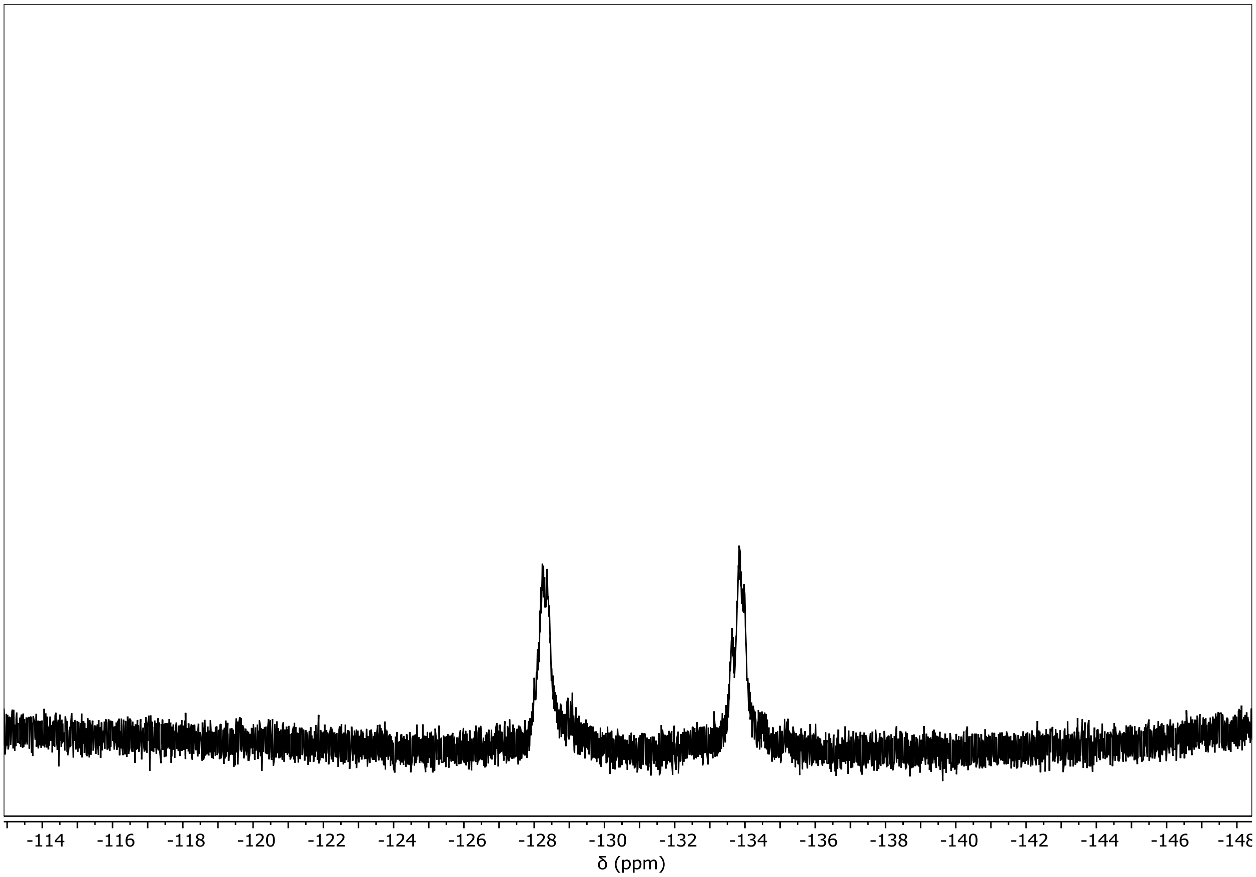

Figure S4. The ^{19}F NMR spectra of **TQ2F-TBTzTD**.

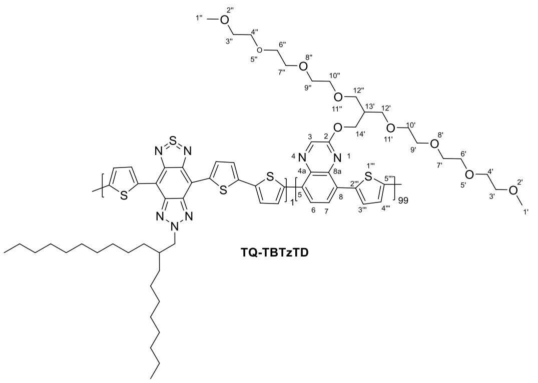

^{1}H NMR (600 MHz, $CDCl_3$) δ 8.70 – 8.59 (1H, m, H3), 8.31 – 8.10 (1H, m, H7), 8.07 – 7.97 (1H, m, H6), 7.97 – 7.72 (2H, m, H3''', H4'''), 4.75 (2H, d, J = 51.8 Hz, H14'), 3.72 – 3.35 (28H, m, H3', H3'', H4', H4'', H6', H6'', H7', H7'', H9', H9'', H10', H10'', H12', H12''), 3.31 – 3.22 (6H, m, H1', H1''), 2.57 – 2.54 (1H, m, H13').

^{13}C NMR (151 MHz, $CDCl_3$) δ 156.7 (C2), 138.0 (C3), 137.3 (C8a), 135.9 (C4a), 132.2 (C2'''), 130.0 (C5'''), 128.2 (C3'''), 128.0 (C4'''), 127.7 (C7), 127.3 (C6), 126.6 (C5), 124.9 (C8), 71.9 (C3', C3''), 71.8 (C9, C9'), 70.7 (C4', C4''), 70.6 (C6', C6''), 70.6 (C12', C12''), 70.5 (C10', C10''), 70.4 (C7', C7''), 69.4 (C14'), 59.0 (C1', C1''), 39.7, 39.5 (C13').

Figure S5. The atomic assignments of **TQ-TBTzTD**, including the derived ^{1}H NMR and ^{13}C NMR peak values.

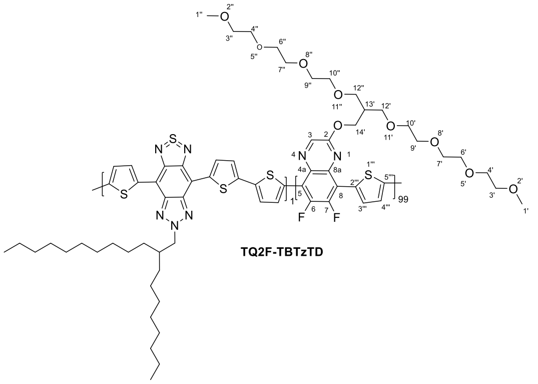

^{1}H NMR (600 MHz, $CDCl_3$) δ 8.78 – 8.50 (1H, m, H3), 8.21 – 7.78 (2H, m, H3''', H4'''), 4.79 – 4.57 (2H, m, H14'), 3.83 – 3.36 (28H, m, H3', H3'', H4', H4'', H6', H6'', H7', H7'', H9', H9'', H10', H10'', H12', H12''), 3.30 – 3.15 (6H, m, H1', H1''), 2.70 – 2.43 (1H, m, H13').

^{13}C NMR (151 MHz, $CDCl_3$) δ 155.9 (C2), 149.3, 147.8 (C7), 147.6, 145.8 (C6), 136.6 (C8a), 134.1 (C3), 133.72 (C4a), 133.7 (C2'''), 133.4 (C5'''), 131.7 (C3'''), 129.8 (C4'''), 119.5 (C5), 116.6 (C8), 70.9 (C3', C3''), 70.8 (C9, C9'), 69.6 (C4', C4''), 69.5 (C6', C6''), 69.5 (C12', C12''), 69.4 (C10', C10''), 68.2 (C7', C7''), 68.2 (C14'), 58.0 (C1', C1''), 38.6 (C13').

Figure S6. The atomic assignments of **TQ2F-TBTzTD**, including the derived ^{1}H NMR and ^{13}C NMR peak values.

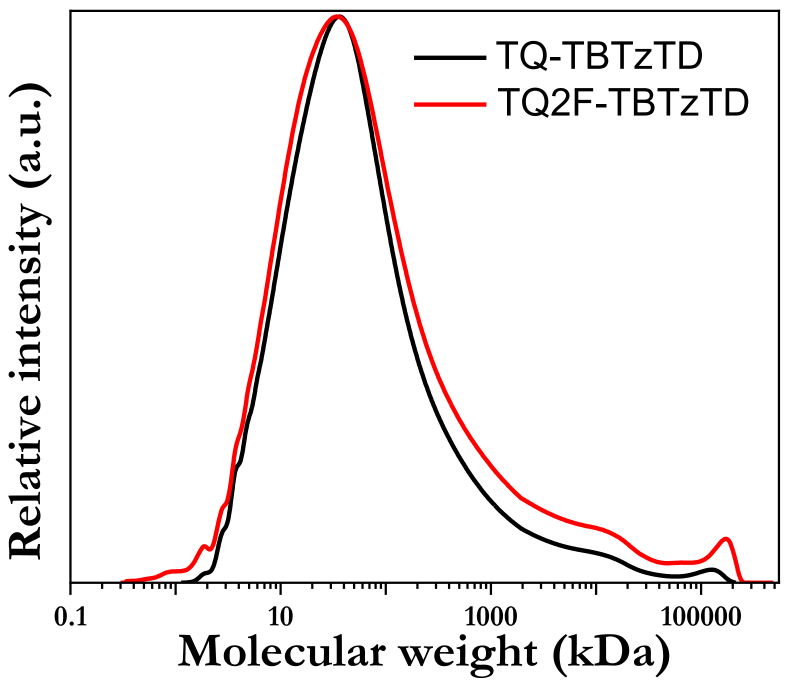

Figure S7. The molecular weight distributions of **TQ-TBTzTD** and **TQ2F-TBTzTD**. The GPC data were recorded at 35 °C using chloroform as the eluent solvent.

The molecular weight distributions in **Figure S7** were measured by gel permeation chromatography (GPC), using polystyrene as the relative calibration standard. The derived values for the number-averaged molecular weight (M_n) are M_n = 22.0 kDa for **TQ-TBTzTD** and M_n = 19.1 kDa for **TQ2F-TBTzTD**.

Both terpolymers thus exhibit a similar degree of polymerization, and we note that the derived values are similar to those previously reported for quinoxaline-based polymers.[4-7] However, it should be noted that both terpolymers appear to exhibit aggregation in the eluent, as implied by the presence of a high molecular weight tail in the chromatograms that extend to unreasonably high values of 100 MDa.[7] Therefore, we have opted to not present the (apparently unreliable) weight-averaged molecular weight (M_w). Moreover, it has been reported that the relative calibration procedure with polystyrene can overestimate the molecular weight, since a rigid conjugated polymer core often exhibits a higher hydrodynamic radii than the flexible polystyrene standard.[8]

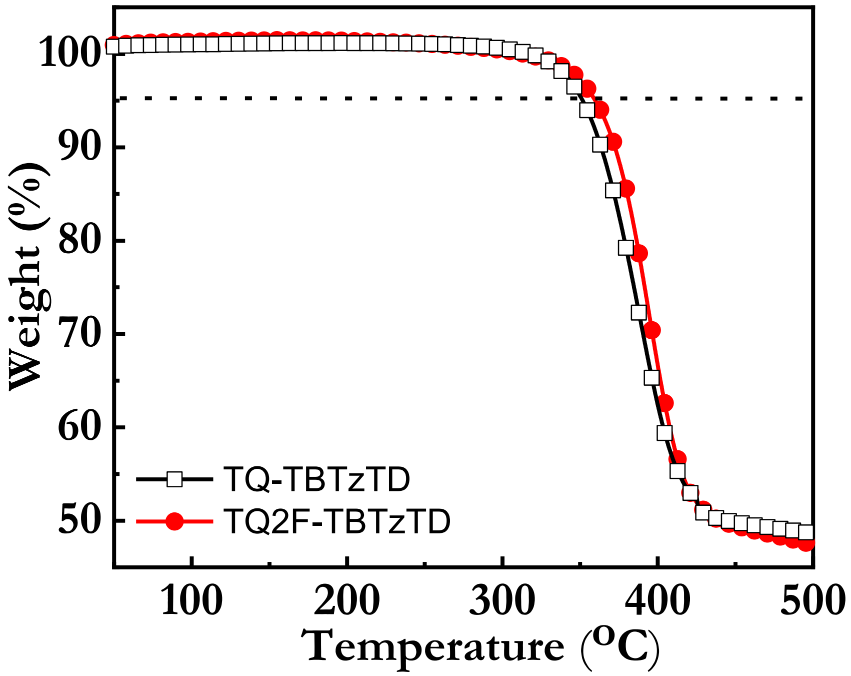

Figure S8. TGA traces of the **TQ-TBTzTD** and **TQ2F-TBTzTD** terpolymers. The scan rate was 10 ^{o}C min^{-1}.

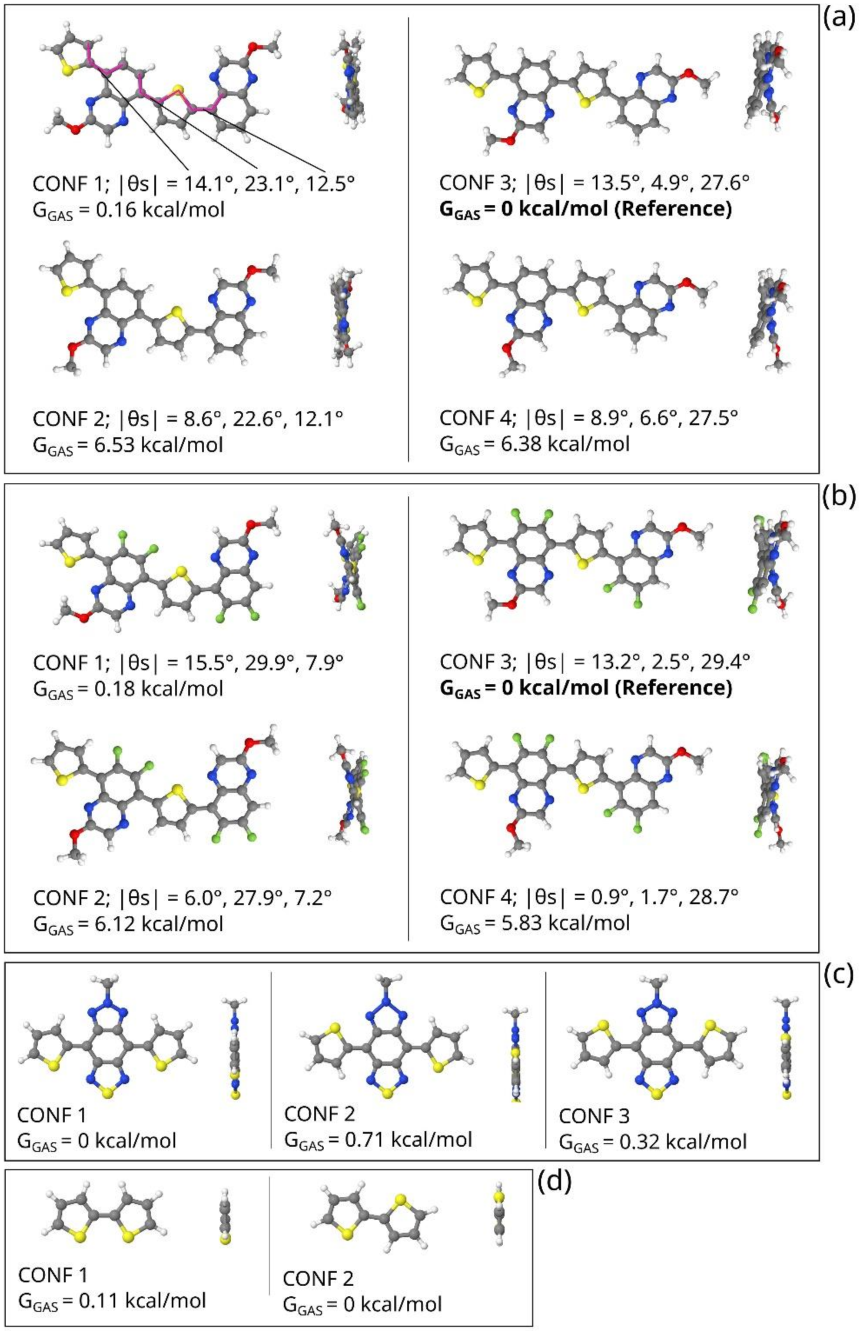

Figure S9. Conformational space exploration for (a, b) the host polymer and (c, d) the guest compounds, as obtained from geometry optimizations of various conformations at the B3LYP/6-311G(d,p) level with GD3BJ dispersion corrections, in gas phase. The conformational stability is assessed via the Gibbs free energy (G), which is calculated as the sum of the electronic energy and thermal corrections. The absence of imaginary frequencies confirmed that each conformation represents a minimum on the potential energy surface. All presented free energies correspond to the most stable conformation, which is set to zero -CONF 3 for the host, CONF 1 for the guest and CONF 2 for the bithiophene.

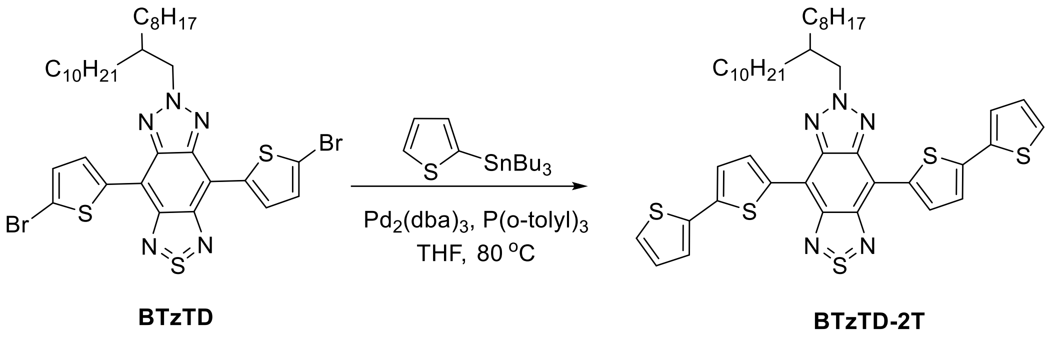

Figure S10. The key step in the synthesis of the NIR-emitting small molecule BTzTD-2T.

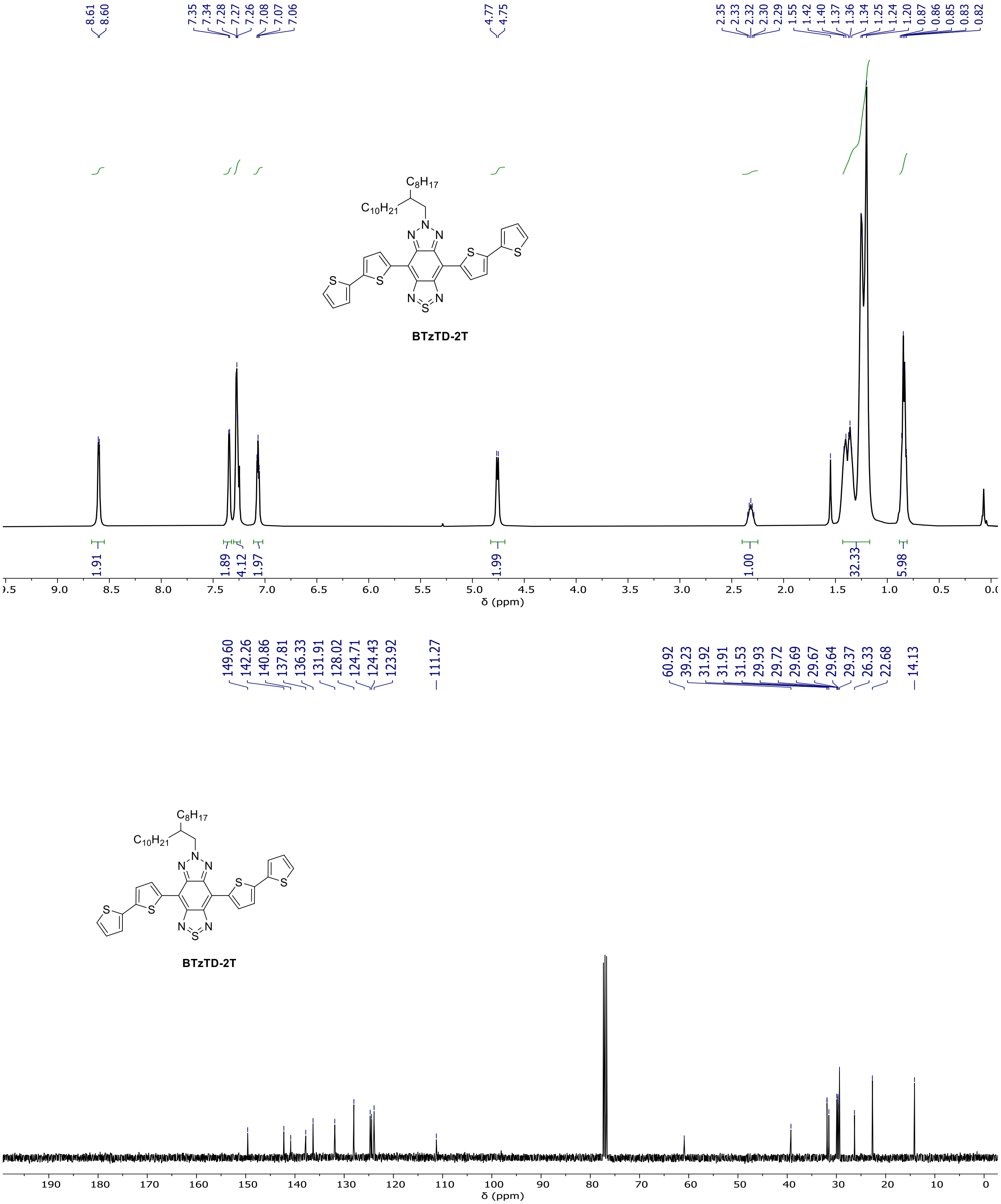

Figure S11. The ^{1}H and ^{13}C NMR spectra of the NIR-emitting small molecule BTzTD-2T.

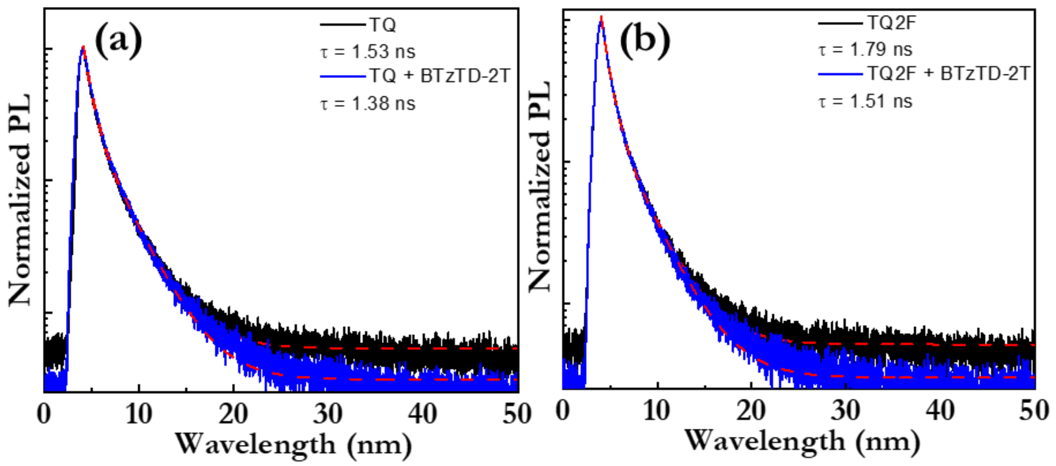

Figure S12. The measured (solid lines) and the fitted (dashed red lines) PL transients of the two host polymers and their blend with the BTzTD-2T guest (1 mass%) in solution, with the host polymer being (a) TQ and (b) TQ2F. The derived PL lifetimes are presented in the insets. The laser excitation wavelength was 337 nm.

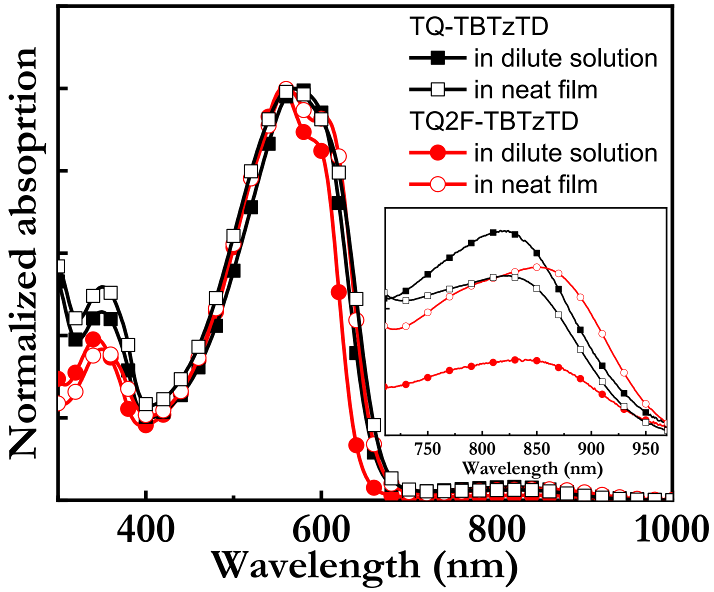

Figure S13. The absorption spectra of **TQ-TBTzTD** and **TQ2F-TBTzTD** in dilute (0.3 g L^{-1}) water:ethanol (v:v=15:85) solution and as a neat film.

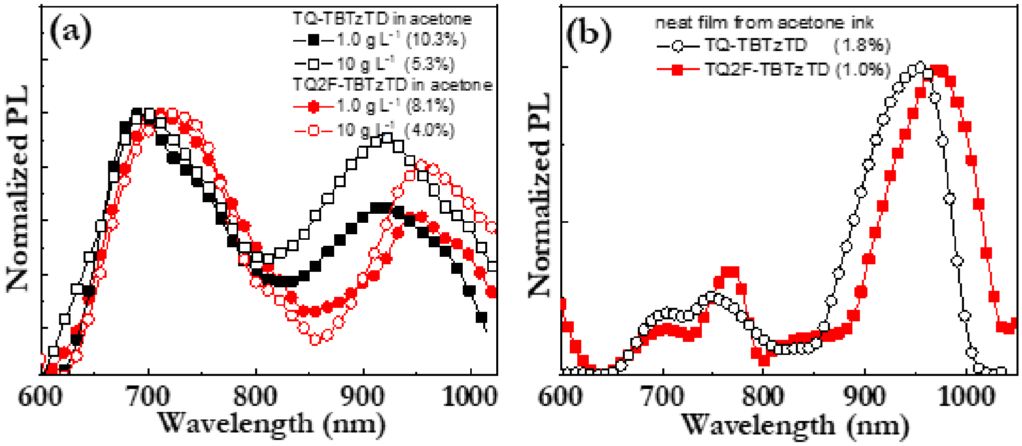

Figure S14. (a) The normalized PL spectra of the terpolymers **TQ-TBTzTD** and **TQ2F-TBTzTD** in acetone. The inset presents the solute concentration and the corresponding values for the PLQY. (b) The PL spectra of neat spin-coated films cast from the acetone solution with a solute concentration of 25 g L^{-1}. The excitation wavelength was 550 nm.

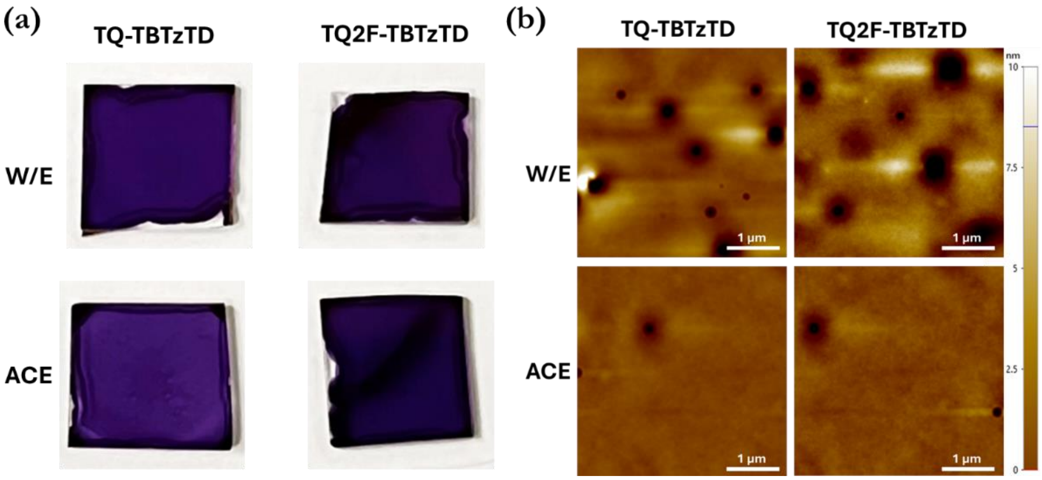

Figure S15. (a) Photographs and (b) AFM micrographs (recorded in non-contact mode) of thin spin-coated films on quartz substrates, with the spin-coating solution being either 25 g L^{-1} acetone (ACE) or the water:ethanol (W/E) blend solution.

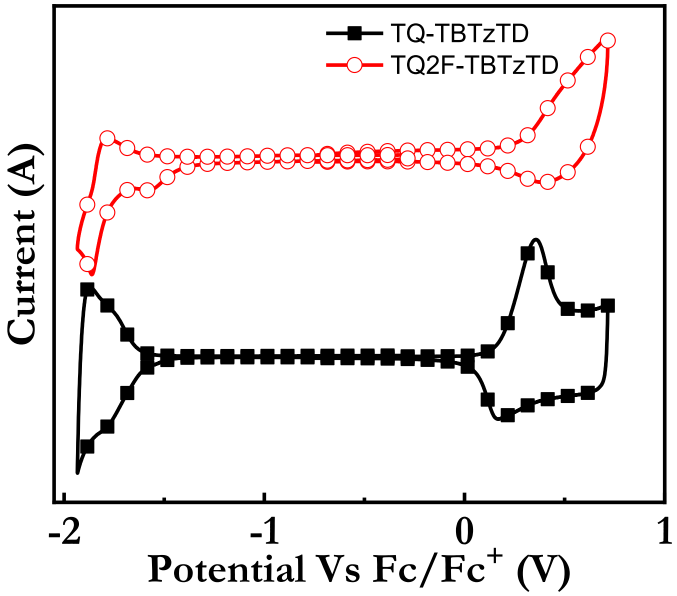

Figure S16. CV traces of neat films of the terpolymers **TQ-TBTzTD** and **TQ2F-TBTzTD**. The scan rate was 0.1 V s^{-1}.

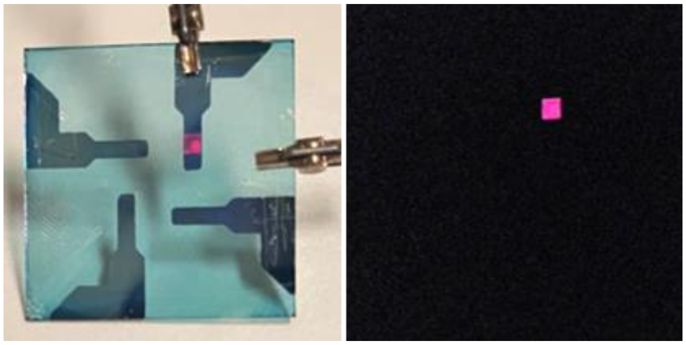

Figure S17. Two photographs of a TQ2F-TBTzTD LEC device with a 2.0×2.0 mm^2 emission area during operation at 4 V, as recorded with (left) no filter and (right) through a filter that only allows wavelength >800 nm to pass through.

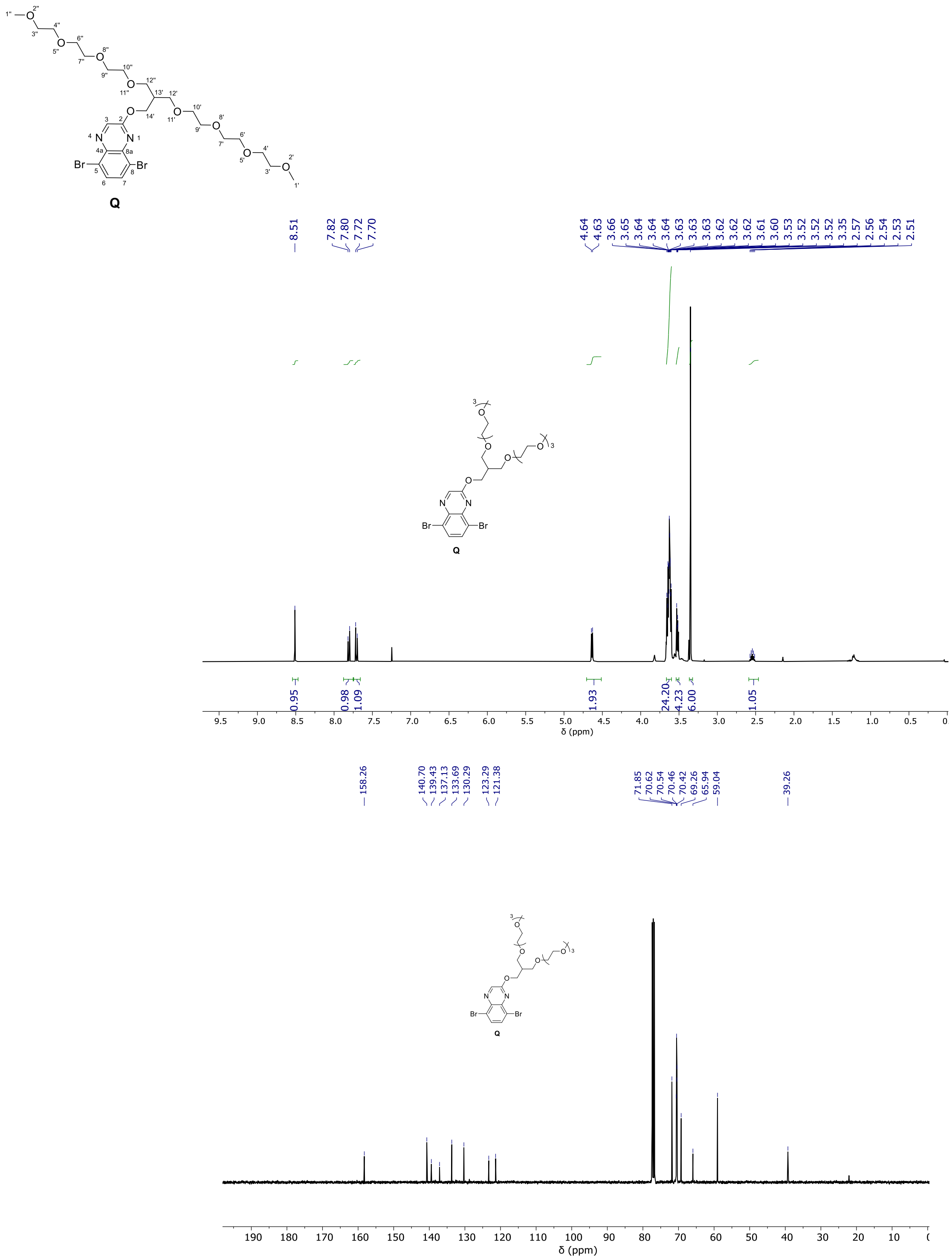

Figure S18. The atomic assignments (upper), the ^{1}H NMR spectrum (middle), and the ^{13}C NMR spectrum (lower) of monomer **Q**.

The monomer 2-((13-(2,5,8,11-tetraoxadodecyl)-2,5,8,11-tetraoxatetradecan-14-yl)oxy)-5,8-dibromoquinoxaline **(Q)** was synthesized following a procedure reported in the literature.[2] Figure

S18 presents the atomic assignments and the ^{1}H NMR and ^{13}C NMR spectra. The derived values for the ^{1}H NMR, ^{13}C NMR and mass spectra peaks are listed below:

^{1}H NMR (400.13 MHz, $CDCl_3$): δ 8.51 (1H, *s*, H3), 7.81 (1H, *d, J* = 8.2 Hz, H7), 7.71 (1H, *d, J* = 8.2 Hz, H6), 4.64 (2H, *d, J* = 5.7 Hz, H14'), 3.66 – 3.60 (24H, *m*, H3', H3'', H4', H4'', H6', H6'', H7', H7'', H9', H9'', H10', H10''), 3.55 – 3.47 (4H, *m*, H12', H12''), 3.35 (6H, *s*, H1', H1''), 2.67 – 2.38 (1H, *m*, H13').

^{13}C NMR (100.6 MHz, $CDCl_3$): δ 158.3 (C2), 140.7 (C3), 139.4 (C8a), 137.1 (C4a), 133.7 (C7), 130.3 (C6), 123.3 (C5), 121.4 (C8), 71.8 (C3', C3''), 70.6 (C9, C9'), 70.5 (C4', C4''), 70.5 (C6', C6''), 70.4 (C10', C10'', C12', C12''), 69.3 (C7', C7''), 65.9 (C14'), 59.0 (C1', C1''), 39.3 (C13').

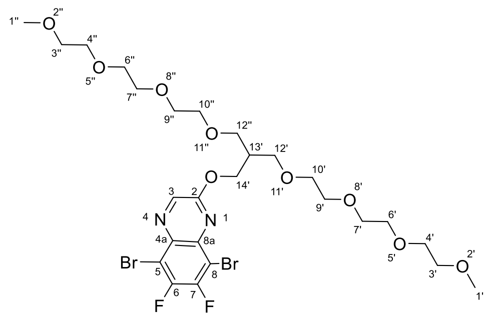

Q2F

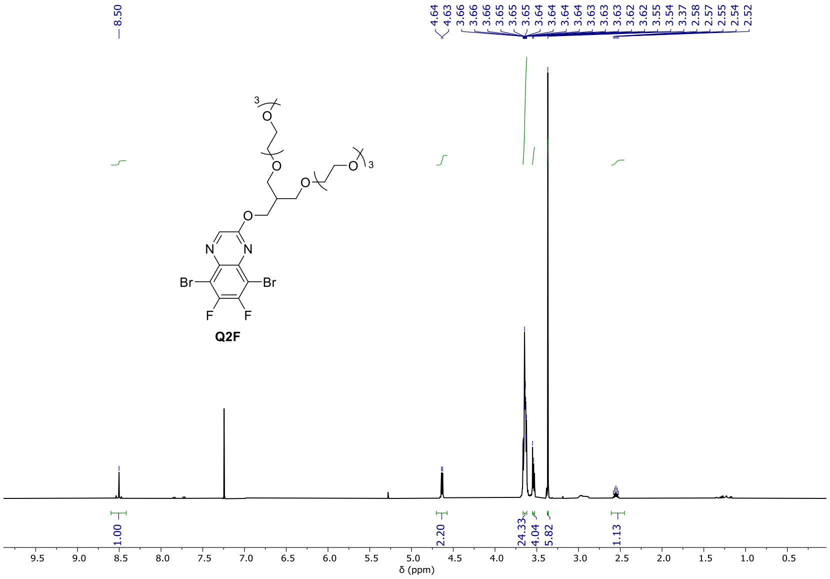

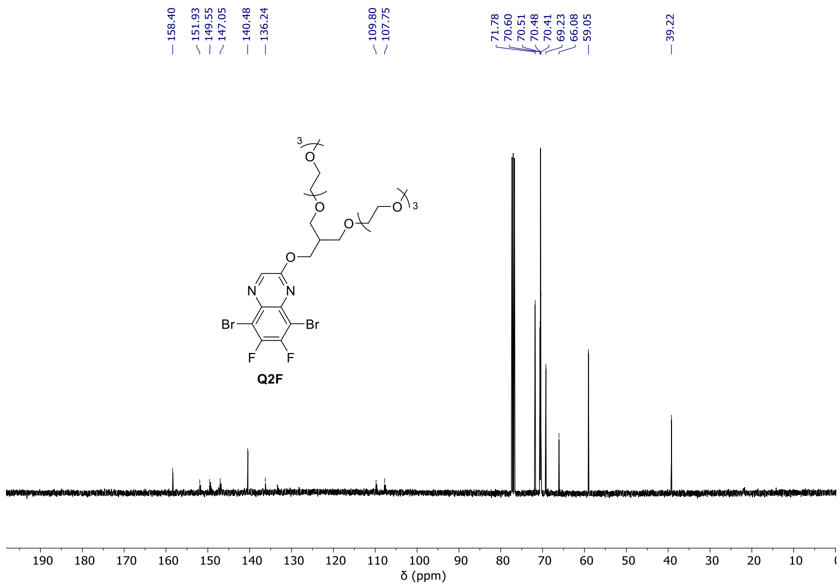

Figure S19. The atomic assignments (upper), the ^{1}H NMR spectrum (middle), and the ^{13}C NMR spectrum (lower) of monomer **QF**.

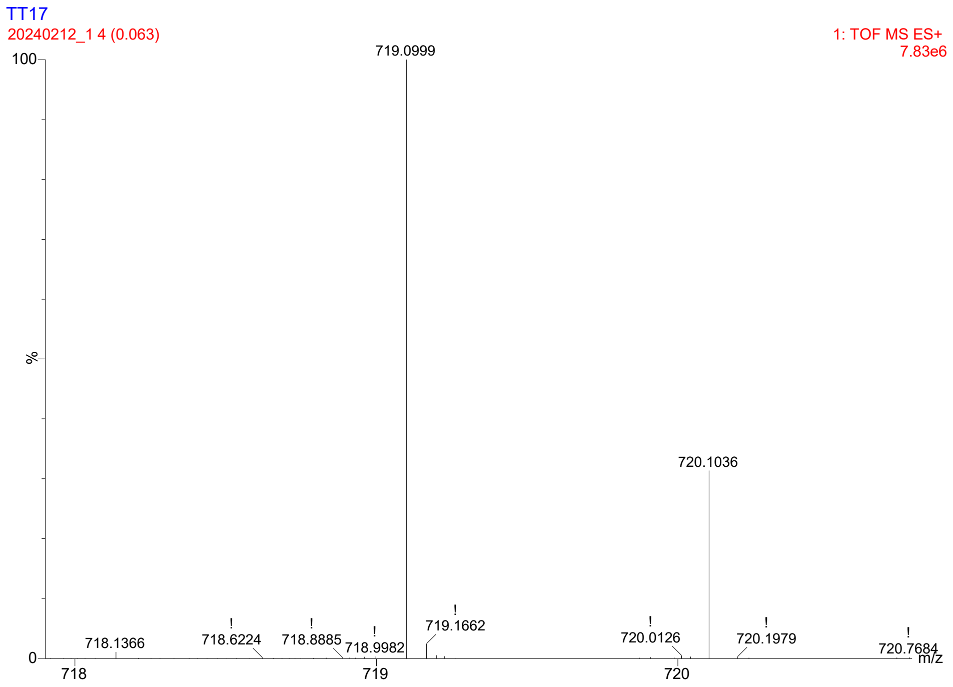

Figure S20. The mass spectrum of monomer **Q2F**, as recorded with a Xevo G2-XS QTof mass spectrometer.

The monomer 2-((13-(2,5,8,11-tetraoxadodecyl)-2,5,8,11-tetraoxatetradecan-14-yl)oxy)-5,8-dibromo-6,7-difluoroquinoxaline **(Q2F)** was synthesized following a procedure reported in the literature.[2, 9]

Figure S19 presents the atomic assignments and the ^{1}H NMR and ^{13}C NMR spectra of **Q2F**, while Figure S20 presents its mass spectrum. The derived values for the ^{1}H NMR, ^{13}C NMR and mass spectra peaks are listed below:

^{1}H NMR (400.13 MHz, $CDCl_3$): δ 8.50 (1H, *s* H3), 4.63 (2H, *d, J* = 5.7 Hz, H14'), 3.77 – 3.59 (24H, *m,* H3', H3'', H4', H4'', H6', H6'', H7', H7'', H9', H9'', H10', H10''), 3.61 – 3.43 (4H, *m,* H12', H12''), 3.37 (6H, *s,* H1', H1''), 2.68 – 2.29 (1H, *m,* H13').

^{13}C NMR (100.6 MHz, $CDCl_3$): δ 158.4 (C2), 151.9 (C7), 149.6 (C6), 147.0 (C3), 140.5 (C8a), 136.2 (C4a), 109.8 (C5), 107.7 (C8), 71.8 (C3', C3''), 70.6 (C9, C9'), 70.5 (C4', C4''), 70.5 (C6', C6''), 70.4(C10', C10'', C12', C12''), 69.2 (C7', C7''), 66.1 (C14'), 59.0 (C1', C1''), 39.2 (C13').

HRMS (ESI+, APCI+) calcd for $C_{26}H_{38}Br_2F_2N_2O_9$ [M+H]+ 719.099, found: 719.0999. FT-IR ν_{max} = 2871, 1706, 1402, 1101, 636 cm^{-1}.

References

1. Murto, P.; Minotto, A.; Zampetti, A.; Xu, X.; Andersson, M. R.; Cacialli, F.; Wang, E., Triazolobenzothiadiazole-Based Copolymers for Polymer Light-Emitting Diodes: Pure Near-Infrared Emission via Optimized Energy and Charge Transfer. *Adv. Opt. Mater.* **2016,** 4, (12), 2068-2076.
2. Filate, T. T.; Tang, S.; Genene, Z.; Edman, L.; Mammo, W.; Wang, E., Hydrophilic Conjugated Polymers for Sustainable Fabrication of Deep-Red Light-Emitting Electrochemical Cells. *Advanced Materials Technologies* **2024,** 9, (3), 2301696.
3. Ilan, Y. A.; Meisel, D.; Czapski, G., The Redox Potential of the O2–O−2 System in Aqueous Media. *Isr. J. Chem.* **1974,** 12, (4), 891-895.
4. Bathula, C.; Song, C. E.; Lee, W.-H.; Lee, J.; Badgujar, S.; Koti, R.; Kang, I.-N.; Shin, W. S.; Ahn, T.; Lee, J.-C.; Moon, S.-J.; Lee, S. K., Synthesis and characterization of quinoxaline-based polymers for bulk-heterojunction polymer solar cells. *Thin Solid Films* **2013,** 537, 231-238.
5. Chen, H.; Zhang, J.; Wang, Q.; Meng, S.; Zhou, Q.; Bai, Y.; Zhang, M.; Ren, Y.; Shen, P.; Xue, L.; Mi, L.; Zhang, Z.-G., Asymmetrically Substituted Quinoxaline Enabling Conformation-Locked Polymer Donors toward High-Performance Polymer Solar Cells. *Chem. Mater.* **2025,** 37, (4), 1541-1553.
6. Barron, J.; Attar, S.; Ghobadi, A.; Gangopadhyay, S.; Sredojevic, D.; Al-Hashimi, M.; Guha, S., Molecularly Engineered Quinoxaline-Pyridyl Pyrazine Polymers for Field-Effect Transistors and Complementary Circuits. *ACS Appl. Electron. Mater.* **2024,** 6, (2), 1464-1474.
7. Lou, X.; van Dongen, J. L. J.; Braeken, Y.; Brebels, J.; van Pruissen, G. W. P.; Li, W.; Wienk, M. M.; Janssen, R. A. J., Superheated high-temperature size-exclusion chromatography with chloroform as the mobile phase for π-conjugated polymers. *Polym. Chem.* **2014,** 5, (2), 558-561.
8. Hayashi, S.; Yamamoto, S.-i.; Koizumi, T., Effects of molecular weight on the optical and electrochemical properties of EDOT-based π-conjugated polymers. *Sci. Rep.* **2017,** 7, (1), 1078.
9. Filate, T. T.; Lee, S.; Franco, L. R.; Chen, Q.; Genene, Z.; Marchiori, C. F. N.; Lee, Y.; Araujo, M.; Mammo, W.; Woo, H. Y.; Kim, B. J.; Wang, E., Aqueous Processed All-Polymer Solar Cells with High Open-Circuit Voltage Based on Low-Cost Thiophene–Quinoxaline Polymers. *ACS Appl. Mater. Interfaces* **2024,** 16, (10), 12886-12896.
